# Circular RNA circPLXNC1 functions as an inhibitor of senescence by regulating autophagy in osteoarthritis

**DOI:** 10.7150/ijbs.114757

**Published:** 2026-01-01

**Authors:** Hongyi Zhou, Zhongyin Ji, Heng Sun, Wanda Zhang, Yizhen Huang, Gu Jin, Haoyu Cai, Xu'an Huang, Jiafeng Dai, Junduo Zhao, Haojie Chen, Shunwu Fan, Yizheng Wu, Jianxiong Shen

**Affiliations:** 1 Department of Orthopedic Surgery, Peking Union Medical College Hospital, Chinese Academy of Medical Sciences and Peking Union Medical College, Beijing, China.; 2 Department of Orthopedic Surgery, Sir Run Run Shaw Hospital, Zhejiang University School of Medicine, Hangzhou, China.; 3 Key Laboratory of Musculoskeletal System Degeneration and Regeneration Translational Research of Zhejiang Province, Hangzhou, China.; 4 Zhejiang Cancer Hospital, Hangzhou Institute of Medicine (HIM), Chinese Academy of Sciences, Hangzhou, China.; H.Z., Z.J. and H.S. contributed equally to this work.

**Keywords:** senescence, circPLXNC1, osteoarthritis, autophagy, ATG7

## Abstract

**Objectives:** Dysfunctional autophagy during aging is a universal inducer of a variety of degenerative diseases. Here, we aim to identify important non-coding RNAs that are crucial for the regulation of osteoarthritis (OA) and age-associated diseases. In addition, we also aim to explore the underlying molecular mechanisms and potential therapeutic strategies.

**Methods:** RNA sequencing based screening was used to identify potential inflammation and autophagy associated key non-coding RNAs. After the identification of targeting molecular circPLXNC1, we examined its expression profile during OA development and in other aging organs and tissues. In addition, knock down of circPLXNC1 both *in vitro* and *in vivo* was used for the functional assays. The downstream targets, miR-130b-5p and ATG7, were also examined. Furthermore, the function of these molecules on autophagy, cell senescence and the progression of OA were also investigated.

**Results:** We identified circPLXNC1 decreased in OA and other aging organs and tissues. CircPLXNC1 acts as a sponge for miR-130b-5p to regulate the expression of autophagy associated enzyme ATG7, which is the direct target of miR-130b-5p. In agreement with the finding that delivery of ATG7 adeno-associated virus (AAV) alleviates OA, the administration of circPLXNC1 AAV exerts similar function to alleviate OA and attenuate the senescence of multiple organs and tissues.

**Conclusions:** We found a potential therapeutic strategy targeting both circPLXNC1 and ATG7 for OA, while circPLXNC1 acted as an inhibitor of senescence in multiple organs and tissues, including cartilage, brain, muscle and adipose.

## Introduction

With the development of medical treatment and social welfare, population proportion of the elderly is gradually increasing. According to the prospect of the Department of Economic and Social Affair of the United Nations, one in every six people will be aged 65 or older by 2050.^1^ The increased elderly population means more attention needed to be paid to age-associated diseases, which occur in different parts of human body and involve multiple organs and tissues, including brain, muscle, adipose and cartilage, leading to a huge social and economic burden.^2^-^7^ During the process of senescence, there are some characteristic phenotype including the induction of senescence-associated secretory phenotype (SASP), altered autophagy, disordered metabolism, cell cycle and morphology changes, chromatin recombination, shorten telomere and so on.^8^-^13^ Neuronal senescence is an important risk factor for the occurrence of Alzheimer's disease and results in increased SASP, accompanied by significantly elevated levels of amyloid beta and abnormally phosphorylated Tau proteins in the neocortex region.^2^ DNA-damage response appears in adipocytes, accelerating adipocyte senescence and genome instability by depletion of SREBP1c.^4^ Similarly, muscle cells release SASP due to depletion of lamin A/C leading not only muscle aging-like deficit, but also osteoporosis.^14^ As for bone diseases, dysregulated SASP appears in senescent osteoarthritis fibroblast-like synoviocytes, enhanced by decreased autophagy flux resulted by METTL3-mediated m6A modification of ATG7.^15^, ^16^ As such, SASP could be seemed as a hallmark of senescence.

Osteoarthritis (OA), the most prevalent age-related degenerative disease, affects more than 240 million people worldwide, accompanied with characteristic pathological changes including cartilage destruction, osteophyte formation, synovial hyperplasia and subchondral bone sclerosis.^17^-^20^ In aging cartilage, the extracellular matrix (ECM), a major component of cartilage, is degenerating due to dysregulation of some enzymes, including COL2A1 and Aggrecan.^20^ Previous studies have demonstrated that aging cartilage in OA is related with metabolic dysregulation, like circRREB1 regulating lipid metabolism related senescent phenotypes, and asymmetric dimethylarginine (ADMA) inducing the degeneration and senescence of chondrocytes.^21^, ^22^ Furthermore, altered epigenetic modification occurs with senescence chondrocytes, such as methylase METTL3 regulating autophagy-GATA4 axis to promote cellular senescence and OA progression.^16^ Mechanistically, some key signaling pathways, Akt signaling, JAK3/STAT5 signaling, NF-κB signaling, Notch signaling and TGF-β/SMAD2/3 signaling, are involved in senescence and OA.^23^-^28^ In addition, some proteins that play significant roles in other tissues also affect OA. For instance, PLXNC1, as a member of the Plexin family, is an important receptor in the nervous system and affects the progression of OA via inducing the expression of GRP78.^29^, ^30^ With the depletion of PLXNC1, the viability injury, inflammation, apoptosis and ECM degradation of chondrocytes exposed to IL-1β were obstructed.^30^ Taken together, these lines of evidence suggest that OA has great research value as an age-associated disease.

Circular RNA (circRNA), a type of endogenous non-coding RNA without a terminal 5' cap and 3′ polyadenylate tail, is produced in eukaryotes by backsplicing with the help of flanking intronic complementary sequences on both sides of certain mRNAs, such as Alu elements, or dimerization of RNA-binding proteins (RBPs), such as FUS and QKI.^31^-^34^ Our previous study revealed that circRNA plays an key role in OA, such as circPDE4D protecting against OA by binding to miR-103a-3p and regulating FGF18, and circARPC1B stabilizing Vimentin to prevent high cholesterol-induced articular cartilage degeneration.^35^, ^36^ Autophagy, as a complex dynamic process related to age-associated diseases, is inhibited in OA by up-regulation of mammalian target of rapamycin (mTOR).^37^, ^38^ At the same time, the absence of autophagy promotes aging and the development of OA 39. Compromised autophagy is also regarded as a hallmark of aging. For instance, autophagy dysregulation in aged mice can lead to neurodegeneration, as well as aging of the heart and skeletal muscles.^40^, ^41^ Meanwhile, the activation of autophagy can delay age-related memory deficits, maintain intestinal barrier function and delay aging in hematopoietic stem cells.^42^ As a manifestation of the protective level of cellular homeostasis and metabolic homeostasis of autophagy, ATG7, the autophagy related protein, can play a role in cytosolic retention of pro-autophagic transcription factors and maintain the benefits of diet for tissues such as adipose and muscle. Meanwhile, conditional ATG7 deficiency leads to a reduction in the muscle satellite cell pool in young mice and causes premature muscle aging.^43^ However, the role of circRNA in regulating senescence and OA by altering autophagy is still in shadow, and its mechanism has not been well elucidated.

As a promising therapeutic platform, researches into circRNA increased rapidly over the past decades. Its high stability and low immunogenicity make circRNA a more suitable therapeutic modality than linear mRNA.^34^, ^44^ AAV, as one of the most common DNA delivery vectors, is often used for circRNA delivery. However, the capacity of AAV genome is less than 4.7kb, limiting its use for the expression of many treatment-related proteins.^44^ The sequence of circRNA is relatively shorter and is more suitable for AAV delivery. For instance, in OA therapy, circPDE4B delivered by AAV abrogates breakdown of cartilage matrix by destabilization of the medial meniscus (DMM) in mice, as well as circARPC1B delivered by AAV reverses high cholesterol-induced osteoarthritis.^45^ Furthermore, circRNAs with distinct roles in pathogenic processes may also translate into clinically useful biomarkers. In cartilage tissue, circUbqln1 expression is negatively correlated with the expression of anabolic markers, including COL2A1 and Aggrecan.^46^ Similarly, circRREB1 is supposed to be characteristically highly expressed in senescence chondrocytes and associated with lipid metabolism.^21^ Nevertheless, how circRNAs used for aging and OA therapy needs further exploration.

In this study, we investigated the function of circPLXNC1 in multiple aging organs and tissues and detailed its mechanism between senescence and OA by regulating autophagy. We believe that our study unshadows a new promising therapeutic strategy for OA and aging.

## Materials and Methods

### OA patient samples

OA patients underwent total knee arthroplasty and provided cartilage tissues. The experimental protocols were approved by the Ethics Committee of Sir Run Run Shaw Hospital. Written informed consents were obtained from all patients. According to the Osteoarthritis Research Society International (OARSI) grading system, Safranin O/Fast Green stain was used to grade human tissues.^47^

### Animal experiments

WT C57BL/6 (B6) mice were purchased from SLAC Laboratory Animal Company (Shanghai, China), whose study protocols were approved by the Ethics Committee of Zhejiang University.

DMM models were created by transecting the medial meniscotibial ligament in 12-week-old male C57BL/6 mice, while sham operation was performed on control mice.^48^ 8 weeks after surgery, the mice would be euthanized. OARSI grade was used to evaluate OA model mice.

After acclimatized to the test room for 30 min, OA model mice were evaluated by animal praxeology assays, including the hot plate test and rotarod test.^35^

HANBIO (Shanghai, China) provided the adeno-associated virus (AAV), which was injected into the knee joint cavity of mice 1 week after DMM surgery at a dose of 5 × 10^9^ PFUs per 10 ml using a 10 μL microsyringe with a 34 G needle (Hamilton Company, Reno, NV, USA). The AAV was also injected into mice by tail vein injection according to the manufacturer's instructions.^17^ As RNA oligo, 5' Cholesterol and 2' OME were used to modify si-mmu-circPLXNC1 in order to enhance its permeability and stability *in vivo*.

### Immunohistochemistry

4% paraformaldehyde was used to fix tissues from mouse and human. After that, 0.5 M EDTA was used to decalcify those tissues. Then they would be embedded in paraffin and sectioned at 3 μm. Xylene was use to deparaffinize the sections and graded ethanol was used to hydrate them. Next, 1% Fast/Green (Sigma‒Aldrich) was used to treat some sections for 3-5 min, followed by 1% acetic acid for 10 s and 1% Safranin O (Sigma‒Aldrich) for 3-5 min. As for immunohistochemistry, 3% hydrogen peroxide (H_2_O_2_) and 5% BSA were used to treated sections, followed by incubation with the indicated antibodies for immunohistochemistry overnight. Horseradish peroxidase (HRP)-conjugated secondary antibodies were used to treat those sections. Finally, DAB (Sigma Aldrich) and hematoxylin (Beyotime) were used to stain them. We scanned and analyzed the sections by the KFBIO scan and analysis system (KFBIO, Zhejiang, China).

### Hematoxylin-Eosin (H&E) staining

The brain, muscle and adipose obtained from young and aging mice was fixed by 4% paraformaldehyde. Then we embedded them in paraffin and sectioned at 4 μm. Those sections were stained with H&E. After that, we used KFBIO scan and analysis system (KFBIO, Zhejiang, China) to scan and analyze the aging phenotype of mice.

### FISH

CircPLXNC1 probes labeled with CY3 was purchased from RiboBio (Guangzhou, China). DAPI (4' ,6-diamidino-2-phenylindole) (Sigma-Aldrich, St. Louis, MO, USA) was used to label cell nuclei. According to the manufacturer's instructions, FISH Kit (RiboBio, Guangzhou, China) detected the probe signals. 3DHISTECH scan and analysis system (3DHISTECH, Budapest, Hungary) was used to obtained images.

### Cell culture

Mouse chondrocytes were isolated from 5-day-old WT mice by 0.2% collagenase digestion. The culture medium consisted of Dulbecco's modified Eagle's medium (DMEM; Gibco, Amarillo, TX, USA), 10% fetal bovine serum (FBS; Gibco) and 1% penicillin/streptomycin. The first passage of chondrocytes was used for analysis. We isolated human articular chondrocytes from the tibial plateaus and femoral condyles of human cartilage. Then they were cultured in the same manner as the mouse chondrocytes. Passage 0 chondrocytes were used for analysis. Crisprbio (Beijing, China) provided C28/I2 normal chondrocytes. We also purchased HEK 293T cells from American Type Culture Collection. DMEM mixed with 10% FBS (Gibco) and 1% penicillin G and streptomycin was used to culture those strains. The environment of cultured cells was humid and contains 5% CO2 and 95% air.

Lentiviruses and plasmids were supplied by HANBIO (Shanghai, China) and Tsingke (Beijing, China). We seeded chondrocytes in 6-well plates and added 1 mL of culture medium to each well. ATG7 and circPLXNC1 lentivirus (1 × 10^7^ PFUs) was used to treated chondrocytes for 12 h. Then polybrene (Yeasen, Shanghai, China) was added into culture medium till the final concentration of 10 μg/mL. Next, that culture medium was used to incubate chondrocytes for another 36 h. After that, fresh culture medium was used to replace the original medium.

### Micromass culture and chondrocyte 3D agarose culture

Micromass cultures was a suitable way to assess ECM deposition in previous studies.^49^, ^50^ We seeded about 150,000 primary mouse chondrocytes at the center of 12-well plates. After culturing for 7 days, 4% paraformaldehyde was used to fix the chondrocytes. Then we stained the chondrocytes by Alcian blue 8 GS (Solarbio, China) at a pH of 0.2. ImageJ software (version 1.53C; National Institute of Health, Bethesda, MD, USA) helped us to quantify the ECM deposition.

In previous studies, Chondrocyte 3D agarose culture was another way to evaluate the ECM deposition as described.^51^ We mixed four volumes of 2.5% agarose with one volume of 5× culture medium and 1× culture medium containing 10% FBS, 5% HEPES, and 5% penicillin/streptomycin to obtain a concentration of 2% agarose. Then the medium was mixed with cell suspension till the concentration was 2 × 106 cells/mL. We added each 24-well plate with 700 μL of the mixture and waited for 15 min till gelling. Besides the mixture, each well was filled with culture medium, which would be replaced daily. After culturing for 14 days, we fixed the hydrogels with 4% paraformaldehyde and embedded them in paraffin. Next, we sectioned them at 7 μm and used Alcian blue to stain them.

### Western blot analysis and antibodies

We mixed RIPA lysis buffer (Fudebio, Hangzhou, China) with protease inhibitor cocktails (Fudebio) and used them to lyse the cells and obtain protein. Fudebio Bicinchoninic Acid protein assay kit was used to detect the concentration of protein. After 45 min of SDS‒PAGE, we would transfect equivalent amounts of protein to 0.22-μm PVDF membranes (Merck KGaA) at 290 mA for 100 min. Then 5% BSA was used to block the membranes for about 1 h. After that, high-affinity antibodies were used to incubate with the membranes. We used TBST to wash them and incubated them with horseradish peroxidase-conjugated secondary antibodies (FDM007 and FDR007, Fudebio). Finally, the antibody signals would be detected by the enhanced chemiluminescence kit (FD8030, Fudebio).

The following antibodies were used for Western blot analysis: anti-β-actin (3700, Cell Signaling Technology [CST], Danvers, MA, USA), anti-COL2A1 (M2139, Santa Cruz Biotechnology, Dallas, TX, USA), anti-Aggrecan (C8035, Millipore, Burlington, MA, USA), anti-SOX9 (ab185966, Abcam), anti-ATG7 (ab133528, Abcam), anti-FUS (11570-1-AP, Proteintech, Rosemont, IL, USA), anti-p16^INK4a^ (ab211542, Abcam), anti-p21 (ab109199, Abcam), anti-p62 (18420-1-AP, Proteintech, Rosemont, IL, USA), anti-LC3B (43566, Cell Signaling Technology [CST], Danvers, MA, USA), and anti-AGO2 (67934-1-AP, Proteintech, Rosemont, IL, USA).

### RNA isolation and RT‒PCR

The RNAEX reagent (Accurate Biotechnology, Hunan, China) and the SteadyPure Universal RNA Extraction Kit (Accurate Biotechnology) were used to extract total RNA. Then Evo M-MLV RT Premix for qPCR (Accurate Biotechnology) was used to generate cDNA through reverse transcription. According to the manufacturer's instructions, HieffR qPCR SYBR Green Master Mix (Yeasen, Shanghai, China) was used for RT‒PCR on the QuantStudioTM 6 Flex Real-Time PCR System (Thermo Fisher Scientific, USA). We used β-actin, a housekeeping gene, as an internal control. The primers were purchased from Tsingke (Beijing, China).

### mRNA stability

We treated human chondrocytes with Actinomycin D (MCE, China, 2 μg/mL), which could inhibit further RNA synthesis. 2 h later, we extracted total RNA from human chondrocytes. Then we calculated remaining mRNA by quantitative RT‒PCR and normalized them to the first group (0 h).

### Cell Counting Kit-8 (CCK-8) assay

Human chondrocytes were cultured in 96-well plate. And we treated the human chondrocytes with Rapamycin at different concentrations. After culturing 48 h, fresh medium containing 10% CCK-8 solution (Yeasen, Shanghai, China) replaced the original cell medium and incubated at 37 °C for 1-4 h. The MultiskanTM FC System (Thermo Fisher Scientific) measured OD 450 nm of each well.

### Luciferase reporter assays

We constructed circPDE4D WT reporter plasmid and mutated the predicted binding site of miR-130b-5p in circPLXNC1 to obtain circPLXNC1 Mut reporter plasmid. Then we transfected HEK 293T cells with the luciferase reporter plasmids by Lipofectamine 3000 transfection reagent (Thermo Fisher Scientific, USA). At the same time, miR-130b-5p mimics or negative control was cotransfected to HEK 293T cells. Similarly, ATG7 3' UTR WT or ATG7 3' UTR Mut plasmid was constructed and transfected to HEK 293T cells with miR-130b-5p mimics or negative control. After 24 h of incubation, the dual-luciferase reporter assay system (Promega, Madison, WI, USA) was used to measure luciferase activity. Relative luciferase activity was calculated by using the value of hRLuc standardized to hFLuc.

### RNA binding protein immunoprecipitation (RIP) assay

According to the manufacturer's instructions, RNA-binding protein immunoprecipitation (RIP) assay kit (Bersinbio, China) was used to perform RIP analysis. After shearing mRNAs to the optimal fragment length via ultrasonication, C28/I2 lysates treated with protease and RNase inhibitors and incubated with anti-FUS antibody (11570-1-AP, Proteintech, Rosemont, IL, USA) overnight. Then we used qRT‒PCR to examine the mRNAs screened by the antibody and magnetic beads. Similarly, C28/I2 lysates treated with protease and RNase inhibitors and incubated with anti-AGO2 (67934-1-AP, Proteintech, Rosemont, IL, USA) overnight. qRT‒PCR examined the mRNAs screened by the antibody and magnetic beads.

### RNA sequencing

Three groups of human chondrocytes isolated from three different patients were cultured to P4 generation. Then they were treated with Rapamycin or not. After that, the three groups treated with Rapamycin were mixed together, while the three groups not treated with Rapamycin were also mixed together. RNAEX reagent (Accurate Biotechnology, Hunan, China) was used to extract total RNA from human chondrocytes in P4 generation treated with Rapamycin or not. LC-Bio Technology (Hangzhou, China) reverse transcribed mRNA into cDNA and made PCR amplification. 2× 150-bp paired-end sequencing (PE150) was performed by the Illumina NovaSeq 6000 (LC-Bio Technology, Hangzhou, China). According to the criteria, that the fold change range was >8 and the P value was <0.01, we screened the meaningful mRNA. Similarly, C28/I2 treated with sh circPLXNC1 or sh NC was used to screened meaningful mRNA according to the same way.

### Statistics

We used GraphPad Prism (version 8; San Diego, CA, USA) to analyze the data and presented them as the means ± SDs. Significant differences between two groups were compared by Student's t test, while differences between multiple groups were calculated by one-way ANOVA. Statistical significance was set at *p < 0.05, **p < 0.01, ***p<0.001, and ****p <0.0001.

## Results

### SASP increases in multiple organs and tissues of aging mouse model with impaired autophagy

**Figure 1:**
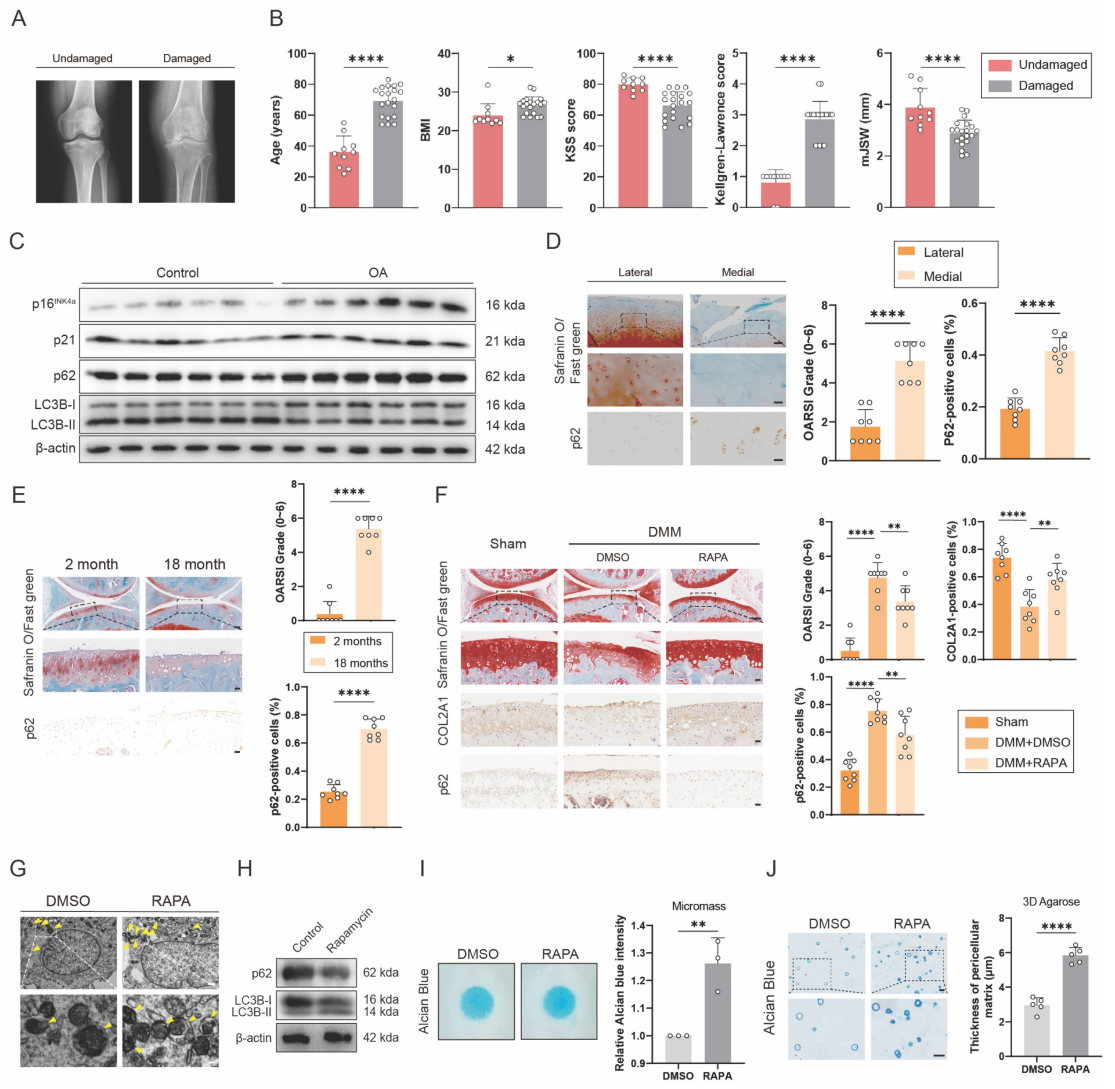
SASP increases in cartilage tissues of aging mouse model with impaired autophagy. (A) Representative knee radiographs of patients with undamaged and damaged knee. (B) Clinical data of patients including age, BMI, Knee Society Score (clinical, KSS), Kellgren-Lawrence (KL) score and minimal medial joint space width (mJSW). (C) Western blot detection of p16^INK4a^, p21, p62 and LC3B expression in human primary chondrocytes from the damaged cartilage group or the undamaged cartilage group. (D) Safranin O/Fast Green staining and immunohistochemistry (p62) of the lateral and medial tibial plateaus of OA patients. n=8. Scale bar, 100 μm and 20 μm. (E) Safranin O/Fast Green staining and immunohistochemistry (p62) of the knee joints of 2-month old and 18-month old mice. n=8 per group. Scale bars, 100 μm and 20 μm. (F) Safranin O/Fast Green staining and immunohistochemistry (FTO) of the knee joints of mice 8 weeks after DMM surgery with treatment of Rapamycin or DMSO or in the sham group. n=8 per group. Scale bars, 100 μm and 20 μm. (G) The representative electron microscopy images of C28/I2 treated with Rapamycin or not. Yellow arrowheads indicate autophagosomes. Scale bars, 5 μm and 1 μm. (H) Western blot detection of p62 and LC3B in C28/I2 treated with Rapamycin or not. (I) Chondrogenic matrix deposition (Alcian Blue staining) of Rapamycin-treated C28/I2 determined by micromass culture and quantified by ImageJ software. (J) 3D agarose culture of C28/I2 (Alcian blue staining) showing the thickness of pericellular matrix. Scale bar, 20 μm. The data are representative of three independent experiments (H). *p<0.05, **p<0.01, ***p<0.001, ****p<0.0001, mean ± SD, two-tailed t test.

To detect the relationship between senescence and autophagy in OA, we collected cartilage samples from OA patients who underwent total knee arthroplasty and patients with knee fractures but no history of OA. These samples were divided into undamaged and damaged groups. Between the two groups, obvious differences were found in age, BMI, KSS score, Kellgren-Lawrence score and mJSW, which were characteristic signals for OA (Figure 1A and B). Western blots showed that expression of senescence marker, p16^INK4a^ and p21, and autophagy marker, p62, increased significantly, while the ratio of LC3B-II to LC3B-I, another autophagy marker, decreased in OA group, compared with control group (Figure 1C and S1J). The medial group had higher OARSI scores and higher p62 expression levels than the lateral group from the same OA patient (Figure 1D). p62 expression was also measured in DMM-induced OA model mice (Figure 1F). The DMM group had higher OARSI scores and higher p62 expression (Figure 1F). Similarly, OARSI scores and p62 expression were higher in 18-month-old mice than in 2-month-old mice (Figure 1E). These findings revealed that senescence marker was higher and autophagy flux was lower in human OA cartilage sections and mouse OA models. Additionally, 18-month-old and 2-month-old mice were used to detect the relationship between senescence and autophagy in other organs and tissues. H&E staining showed obvious aging phenotype of 18-month-old mice compared to 2-month-old mice in brain, muscle and adipose (Figure S1A). At the same time, the expression of p16^INK4a^ and p62 was higher in the brain of 18-month-old mice than in the brain of 2-month-old mice (Figure S1B). Similarly, the IHC results of muscle and adipose showed the same trend in 18-month-old and 2-month-old mice (Figure S1C and E).

To further detect the effect of autophagy on OA, Rapamycin, which could increase autophagy flux, was used to inject into the articular cavity of mice suffering DMM surgery. Safranin O staining showed that mice treated with Rapamycin had lower OARSI scores than the group treated with DMSO (Figure 1F). The results of IHC verified that p62 expression decreased while COL2A1 expression increased after using Rapamycin (Figure 1F). In addition, transmission electron microscope (TEM) was used to detect the autophagosomes, a marker of autophagy, which increased in C28/I2 treated with Rapamycin of 5 μM concentration (Figure 1G and Figure S1F and G). Western blots also showed that the expression of p62 decreased and the ratio of LC3B-II to LC3B-I increased in C28/I2 after treated with Rapamycin (Figure 1H and S1K). Conversely, the expression of p62 and LC3B showed different tendency in C28/I2 after treated with 3-MA, which could reduce autophagy flux (Figure S1H). In addition, western blots showed that the expression of p16^INK4a^ and p21 decreased in C28/I2 after treated with Rapamycin (Figure S1I). What's more, micromass culture and 3D agarose culture of C28/I2 revealed that extracellular matrix (ECM) deposition increased in C28/I2 treated with Rapamycin (Figure 1I and J). Taken together, ECM expression increases with the increase of autophagy flux.

### CircPLXNC1 is downregulated in OA and is associated with aging and autophagy

**Figure 2:**
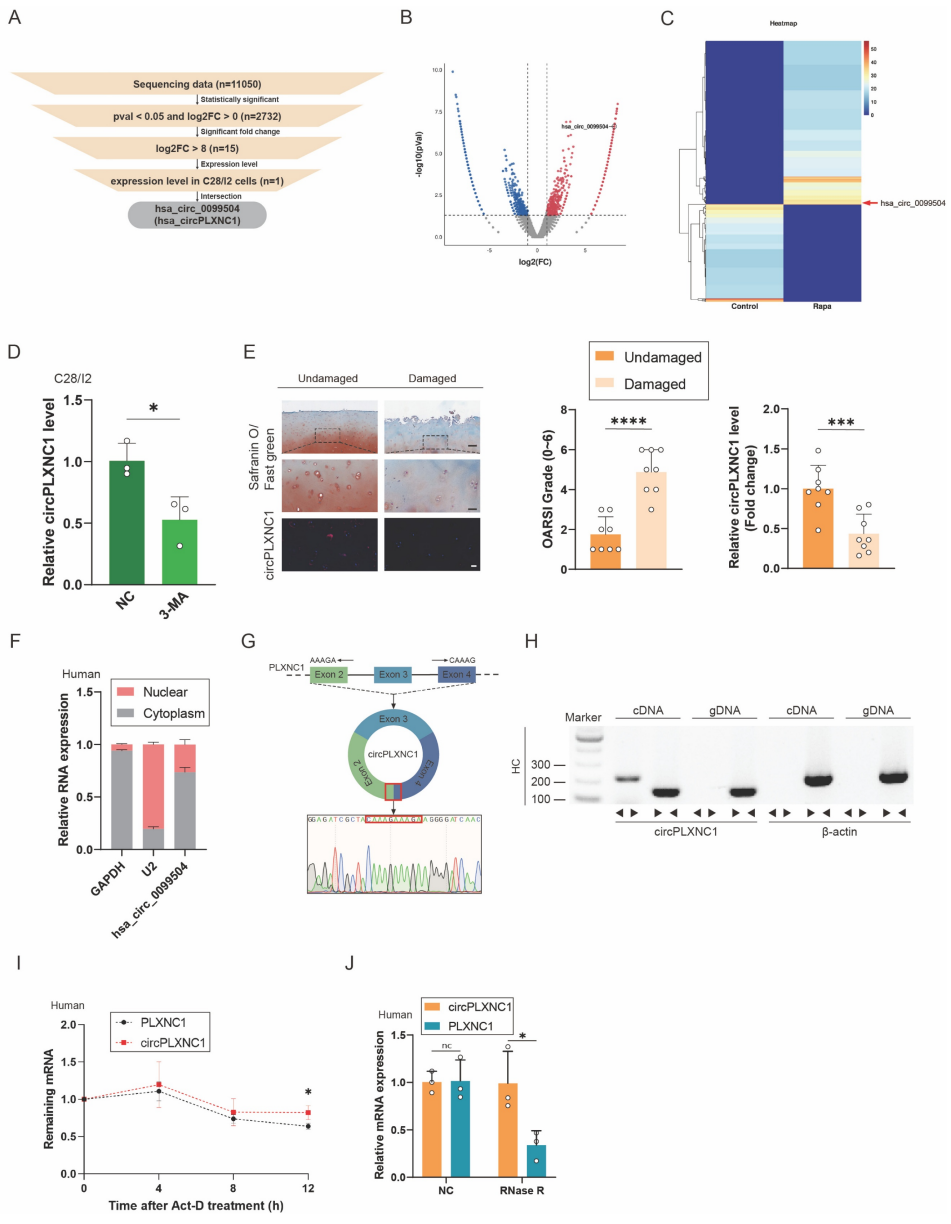
CircPLXNC1 is downregulated in OA and is associated with aging and autophagy. (A) The flowchart illustrating the selecting processes of circPLXNC1 based on the sequencing data. (B) Volcano plot of RNA-seq of human chondrocytes from P4 generation treated with Rapamycin or not. (C) Heatmap of circRNA from human chondrocytes from P4 generation treated with Rapamycin or not. (D) mRNA expression levels of circPLXNC1 in C28/I2 treated with 3-MA or not. (E) Representative images of RNA fluorescence *in situ* hybridization (FISH) and Safranin O/Fast Green staining of undamaged and damaged regions from the lateral and medial tibial plateaus of OA patients. n=8. Scale bar, 100 μm and 20 μm. (F) Expression of circPLXNC1 assessed by RT-qPCR in the nuclear and cytoplasmic fractions of human chondrocytes. (G) Schematic illustration demonstrating the circularization of PLXNC1 exon 2-4 to form circPLXNC1. The presence of circPLXNC1 was validated by RT-PCR followed by Sanger sequencing. The red frame represents “head-to-tail” circPLXNC1 splicing sites. (H) The presence of circPLXNC1 in human HCs was validated by RT-PCR. Divergent primers amplified circPLXNC1 from cDNA but not from genomic DNA; β-actin served as the negative control. (I) The levels of circPLXNC1 and PLXNC1 in HCs treated with actinomycin D at the indicated time points were detected by qRT-PCR. (J) The expression of circPLXNC1 and linear PLXNC1 mRNA in HCs treated with or without RNase R was detected by qRT-PCR. The relative levels of circPLXNC1 and PLXNC1 mRNA were normalized to the value obtained with the mock treatment. *p<0.05, **p<0.01, ***p<0.001, ****p<0.0001, mean ± SD, two-tailed t test.

To investigate the role of circRNAs in senescent human chondrocytes with the change of autophagy flux, human primary chondrocytes extracted from three different knee cartilage tissue were cultured to P4 generation respectively, and then, were treated with Rapamycin or not (Figure S2D and E). After that, human chondrocytes treated with or without Rapamycin were mixed into two groups. RNA sequence was performed on the two groups of primary chondrocytes to examine transcript expression of circRNAs. Significant changes of transcript expression between the two groups were determined by log2-fold change ≥ 0 and *P*-value < 0.05. A total of 11050 circRNAs were detected from the sequencing data, and 2732 circRNAs transcripts were differentially expressed. After that, we selected 15 circRNAs with log2-fold change ≥ 8 from screening results of the previous step. In addition, qRT-PCR was used to verified the expression change of those 15 circRNAs. Among them, 5 circRNAs changed significantly after treatment with IL-1β, which could simulate the inflammatory microenvironment in OA. On the other hand, 4 circRNAs changed significantly after treatment with rapamycin. After the intersection of those two results, the screening result circPLXNC1 (has_circ_0099504) was obtained (Figure 2A-C and Figure S2A and B). What's more, qRT-PCR was used to detected the expression of circPLXNC1 in primary human chondrocytes treated with 3-MA, which is an inhibitor of autophagy. CircPLXNC1 expression was lower in chondrocytes treated with 3-MA than control group (Figure 2D). Furthermore, IHC revealed that the damaged group had higher OARSI scores and lower circPLXNC1 expression (Figure 2E). Through these experiments, we identified that circPLXNC1 expression was reduced under low autophagy flux condition and OA condition. Additionally, FISH and IHC results of 18-month-old and 2-month-old mice showed that circPLXNC1 expression was lower in aging mice, while the expression of p16^INK4a^ and p62 was higher in aging mice, which indicated that circPLXNC1 expression was lower in brain, muscle and adipose in aging mice than young mice and the autophagy flux was lower in aging mice than young mice as well. (Figure S1B-E).

To explore the characteristics of circPLXNC1, we detected its expression in nucleus or cytoplasm. FISH and qRT-PCR indicated that circPLXNC1 was predominantly localized in the cytoplasm (Figure 2E and F and Figure S2C). To detect the circular nature of circPLXNC1, we designed convergent primers and specific divergent primers for PDE4D mRNA and circPDE4D. The result of PCR showed that circPLXNC1 could be amplified by the divergent primers from cDNA but not from gDNA, whereas PLXNC1 mRNA was detected in both the cDNA and gDNA samples (Figure 2G and H). To compare the resistance degree of degradation of liner RNA and circPLXNC1, actinomycin D and RNase R were used to treat human chondrocytes. qRT-PCR verified that circPLXNC1 degraded slower than PLXNC1 and its expression did not change significantly while the expression of PLXNC1 decreased after treated with actinomycin D or Rnase R, which indicated that circPLXNC1 was more stable (Figure 2I and J). These results demonstrated the critical characteristics of circPLXNC1.

### CircPLXNC1 regulates senescence and autophagy in OA chondrocytes

**Figure 3:**
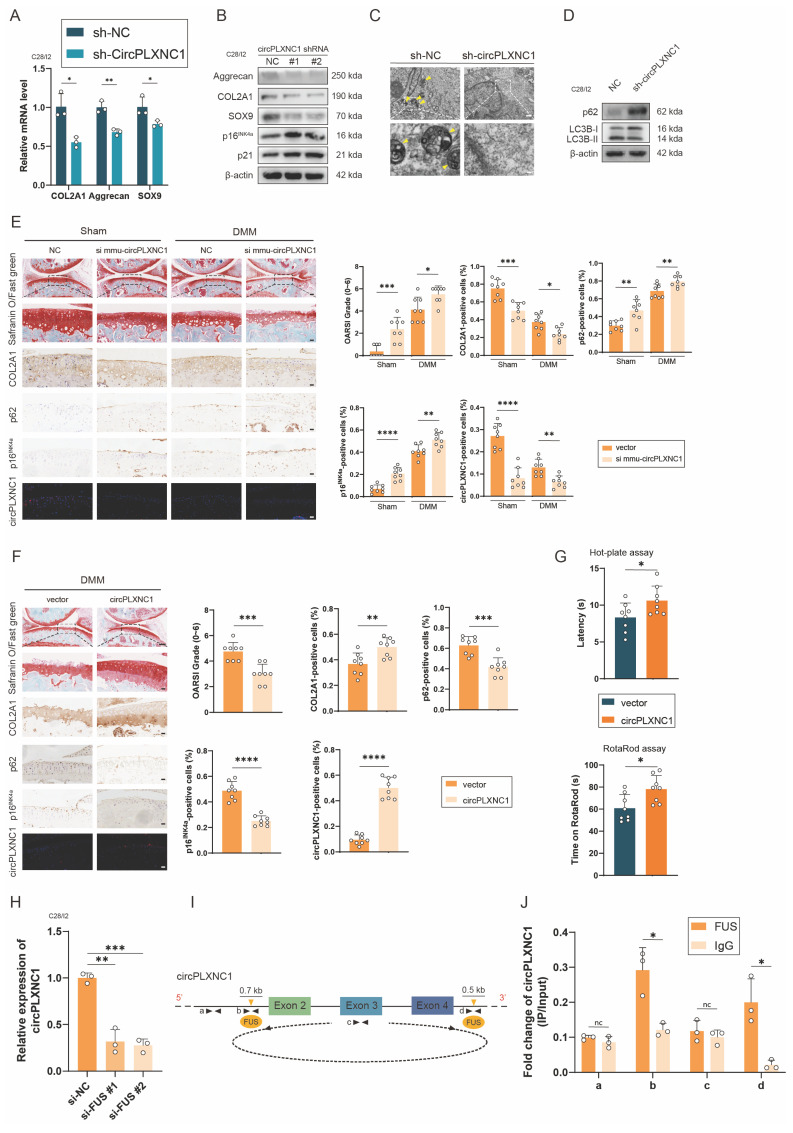
CircPLXNC1 regulates senescence and autophagy in OA chondrocytes. (A) The mRNA expression level of COL2A1, Aggrecan and SOX9 in C28/I2 treated with sh-circPLXNC1 (n=3) or sh-NC (n=3). (B) Western blot detection of COL2A1, Aggrecan, SOX9, p16^INK4a^ and p21 in C28/I2 treated with sh-circPLXNC1 (n=3) or sh-NC (n=3). (C) The representative electron microscopy images of C28/I2 treated with sh-circPLXNC1 or sh-NC. Yellow arrowheads indicate autophagosomes. Scale bars, 5 μm and 1 μm. (D) Western blot detection of p62 and LC3B in C28/I2 treated with sh-circPLXNC1 or sh-NC. (E) Safranin O/Fast Green staining, immunohistochemistry (COL2A1, p62 and p16^INK4a^) and RNA fluorescence *in situ* hybridization (FISH) of the knee joints of the mice that underwent DMM surgery or the sham group. Articular injection of si mmu-circPLXNC1 or vector was administered. n=8 per group. Scale bars, 100 μm and 20 μm. (F) Safranin O/Fast Green staining, immunohistochemistry (COL2A1, p62 and p16^INK4a^) and RNA fluorescence *in situ* hybridization (FISH) of the knee joints of the mice that underwent DMM surgery. Articular injection of circPLXNC1 adeno-associated virus (AAV) or vector. n=8 per group. Scale bars, 100 μm and 20 μm. (G) The hot plate and rotarod test results of mice that underwent DMM surgery. Articular injection of circPLXNC1 adeno-associated virus (AAV) or vector. (H) The relative mRNA levels of circPLXNC1 after FUS knockdown were detected by qRT-PCR. (I) Schematic model of FUS-mediated circPLXNC1 produced via 2 binding sites (interacting with 'GUGGU' motifs) on the flanked intron regions of circPLXNC1-forming exons. (J) The corresponding mRNAs in (I) were pulled by FUS and IgG and detected by RNA immunoprecipitation assay followed by qRT-PCR. The data are representative of three independent experiments (B and D). *p<0.05, **p<0.01, ***p<0.001, ****p<0.0001, mean ± SD, two-tailed t test.

To investigate the role of circPLXNC1 in the regulation of senescence and autophagy in OA chondrocytes, we designed small hairpin RNAs (shRNAs) of circPLXNC1, which could specifically knock down circPLXNC1 in C28/I2 cells (Figure S3A). Because circPLXNC1 and linear PLXNC1 share a partial sequence, we also tested the effects of sh-circPLXNC1 on linear PLXNC1 mRNA (Figure S3A and D). In C28/I2 translated with sh-circPLXNC1, the transcript and protein expression of anabolic enzymes, including Aggrecan, COL2A1 and SOX9 decreased significantly, whereas the protein expression of senescence marker, p16^INK4a^ and p21, increased significantly (Figure 3A and B and S3H). Besides, we also designed si-PLXNC1 and used it to treat C28/I2 cells. In C28/I2 translated with si-PLXNC1, the transcript expression of Aggrecan increased, while the transcript expression of COL2A1 decreased (Figure S3E). What's more, micromass culture and 3D agarose culture of chondrocytes revealed that extracellular matrix (ECM) deposition decreased in C28/I2 treated with sh-circPLXNC1 (Figure S3B and C). At the same time, the results of TEM showed that the number of autophagosomes, a marker of autophagy, was reduced in C28/I2 treated with sh-circPLXNC1 (Figure 3C). Similarly, protein expression of autophagy markers, p62, increased and another autophagy marker, the ratio of LC3B-II to LC3B-I, decreased significantly (Figure 3D and S3I). These findings suggested that with the knockdown of circPLXNC1, anabolic enzyme expression and autophagy flux decreased, whereas the senescence marker increased.

Because circPLXNC1 and its mouse homolog mmu-circPLXNC1 share very high conservation and homology with 87% sequence similarity, we designed si mmu-circPLXNC1 and adeno-associated virus (AAV) carrying circPLXNC1 to investigate the function of circPLXNC1 *in vivo* (Figure S3G). Wild-type mice were treated with surgical destabilization of the medial meniscus (DMM), inducing OA model, for 1 week and then treated with si mmu-circPLXNC1 or si NC by injecting them into the articular cavity for 7 weeks. Both in the sham groups and DMM groups, Safranin O staining showed that mice treated with si mmu-circPLXNC1 had lower OARSI scores than the NC group (Figure 3E). Immunohistochemistry (IHC) revealed that mice treated with si mmu-circPLXNC1 had lower COL2A1 expression and higher p16^INK4a^ and p62 expression, indicating that the decrease in the expression of COL2A1, and the increase in the expression of p16^INK4a^ and p62 were related to the decrease in circPLXNC1 in DMM-induced OA model mice (Figure 3E). On the other hand, after DMM surgery for 1 week, circPLXNC1 AAV was injected into the articular cavity of wild-type mice. OARSI scores were higher in the group treated with circPLXCN1 AAV than in the group treated with vector, as determined by Safranin O staining (Figure 3F). At the same time, IHC revealed that the expression of COL2A1 was greater in the group treated with circPLXNC1 AAV, whereas the expression of p16^INK4a^ and p62 was lower (Figure 3F). In addition, mice treated with circPLXNC1 AAV were less sensitive to pain and had a lower degree of mobility impairment in the hot plate test and rotarod test (Figure 3G). These findings suggested that in the overexpression of circPLXNC1, anabolic enzyme expression and autophagy flux increased, whereas senescence marker expression decreased.

To have a deeper understanding of circPLXNC1, we explored the formation of circPLXNC1. Splicing factors played an important role in regulating the formation of circRNA by binding to the flanking introns during the alternative splicing. Previous studies have reported that FUS, QKI, DHX9, and ADAR1 are most well-known regulators. However, appropriate Alu elements, which is necessary for the binding of DHX9 and ADAR1, could not be found in 700 bp upstream and 500 bp downstream of the flanking introns closing to circPDE4D. Likewise, QKI response elements (QREs), which is necessary for the binding of QKI, could not be found in the same region. Instead, the motifs of FUS were found in 700 bp upstream and 500 bp downstream of the flanking introns closing to circPLXNC1 (Figure 3I and S3F). In addition, we designed two si-RNAs for FUS. qRT-PCR revealed that circPLXNC1 expression decreased after downregulating FUS (Figure 3H). Furthermore, RNA immunoprecipitation (RIP) analysis confirmed the binding regions of FUS were flanking introns instead of circRNA or its remote introns (Figure 3J).

### CircPLXNC1 functions as a sponge for miRNAs and miR-130b-5p triggers aggravates extracellular matrix degradation and impaired autophagy

**Figure 4:**
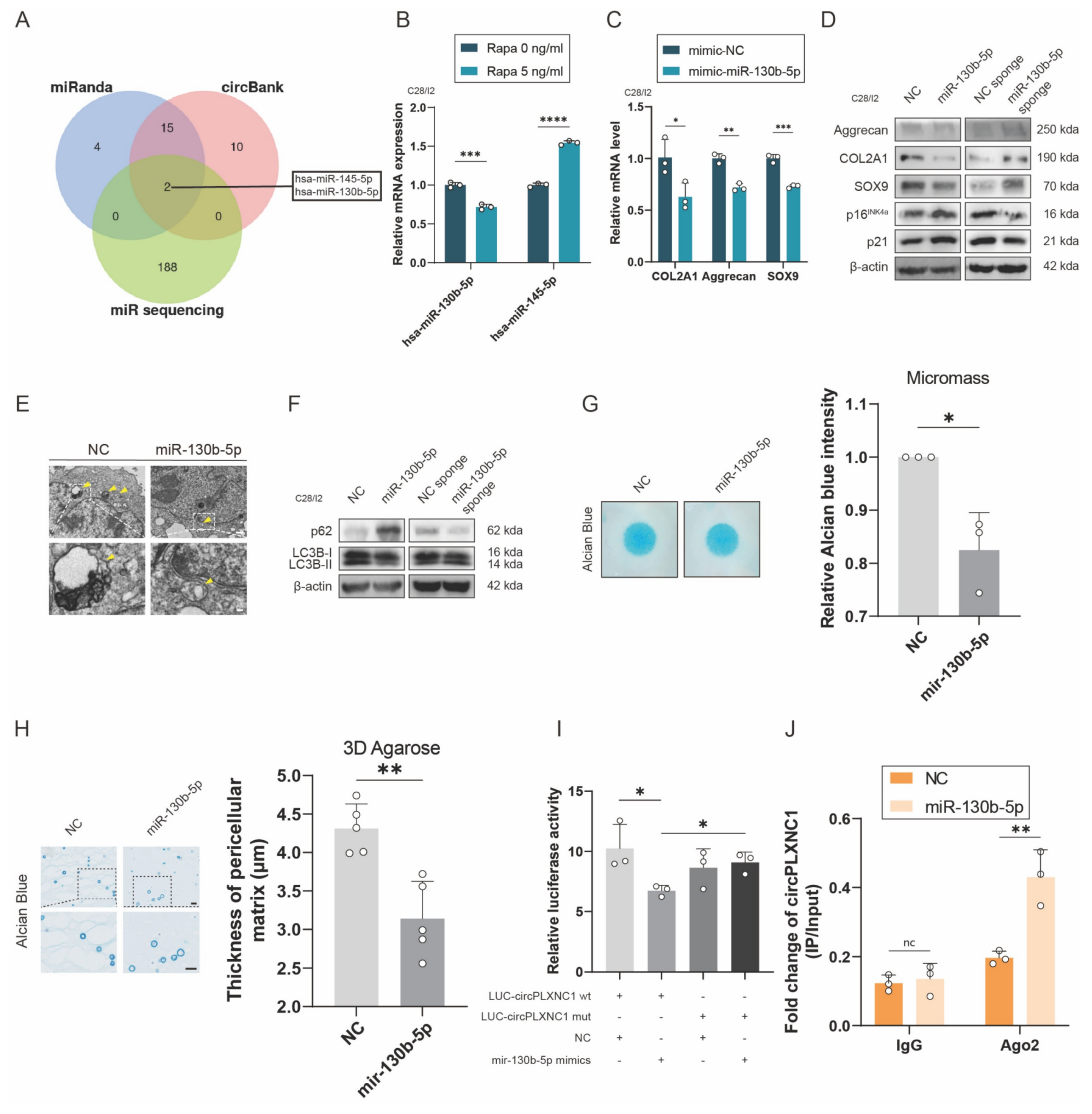
CircPLXNC1 functions as a sponge for miRNAs and miR-130b-5p triggers aggravates extracellular matrix degradation and impaired autophagy. (A) Schematic illustration showing the overlap of the potential target miRNAs of circPLXNC1 predicted using the miRanda and circbank databases and the miRNA sequences. (B) The expression levels of miR-130b-5p and miR-145-5p in C28/I2 treated with Rapamycin (n=3) or not (n=3). (C) The mRNA expression level of COL2A1, Aggrecan and SOX9 in C28/I2 treated with mimic-miR-130b-5p (n=3) or mimic-NC (n=3). (D) Western blot detection of COL2A1, Aggrecan, SOX9, p16^INK4a^ and p21 in C28/I2 treated with mimic-miR-130b-5p (n=3) and mimic-NC (n=3), miR-130b-5p sponge (n=3) or NC sponge (n=3). (E) The representative electron microscopy images of C28/I2 treated with mimic-miR-130b-5p or mimic-NC. Yellow arrowheads indicate autophagosomes. Scale bars, 5 μm and 1 μm. (F) Western blot detection of p62 and LC3B in C28/I2 treated with mimic-miR-130b-5p (n=3), mimic-NC (n=3), miR-130b-5p sponge (n=3) or NC sponge (n=3). (G) Chondrogenic matrix deposition (Alcian Blue staining) of C28/I2, treated with mimic-miR-130b-5p or mimic-NC, determined by micromass culture and quantified by ImageJ software. n=3 per group. (H) 3D agarose culture of C28/I2 (Alcian blue staining) showing the thickness of pericellular matrix. n=5 per group. Scale bar, 20 μm. (I) HEK293T cells were transfected with miR-130b-5p (or NC) and circPLXNC1 reporter (or circPLXNC1 mut reporter) and then used for luciferase activity detection. (J) The circPLXNC1 levels in C28/I2 cells transfected with NC or miR-130b-5p were detected by Ago2 RNA immunoprecipitation assay (RIP assay). IgG was used as a control. The data are representative of three independent experiments (D and F). *p<0.05, **p<0.01, ***p<0.001, ****p<0.0001, mean ± SD, two-tailed t test.

Previous studies have reported that circRNA could act as a sponge to abrogate the function of miRNA. To understand deeper about the role of circPLXNC1 in regulating senescence and autophagy in OA chondrocytes, we used the online software miRanda and circbank and an OA-related RNA sequence to screen potential miRNA regulated by circPLXNC1. miR-145-5p and miR-130b-5p were the two candidate miRNAs obtained by screening (Figure 4A). qRT-PCR was used to detect the expression of miRNAs in human chondrocytes treated by Rapamycin. Only the expression of miR-130b-5p decreased after treated by rapamycin (Figure 4B). Therefore, miR-130b-5p was selected for subsequent studies. In addition, qRT-PCR showed the expression of miR-130b-5p was higher in human primary chondrocytes cultured to P4 generation than P1 generation. Meanwhile, when IL-1β was used to treat C28/I2, the expression of miR-130b-5p increased significantly (Figure S4E and F).

To investigate the role of miR-130b-5p in regulating senescence and autophagy in OA chondrocytes, we designed mimic and inhibitor of miR-130b-5p. In C28/I2 translated with mimic-miR-130b-5p, the transcript and protein expression of anabolic enzymes, including Aggrecan, COL2A1 and SOX9, decreased significantly, whereas the protein expression of senescence marker, p16^INK4a^ and p21, increased significantly (Figure 4C and D and S4I and J). In comparison, the transcript and protein expression of anabolic enzymes, including Aggrecan, COL2A1 and SOX9, increased significantly, whereas the protein expression of senescence marker, p16^INK4a^ and p21, decreased significantly in C28/I2 translated with miR-130b-5p inhibitor (Figure 4D and Figure S4A). In addition, the results of TEM showed that the autophagosomes reduced, as well as the protein expression of autophagy markers, p62, increased and the ratio of LC3B-II to LC3B-I decreased in C28/I2 treated with mimic-miR-130b-5p (Figure 4E and F and S4K). Conversely, under the use of miR-130b-5p inhibitor, the results of TEM and protein expression of autophagy markers were opposite to those under the use of mimic-miR-130b-5p (Figure 4F and Figure S4B). What's more, micromass culture and 3D agarose culture of C28/I2 revealed that extracellular matrix (ECM) deposition decreased in C28/I2 treated with mimic-miR-130b-5p, but increased in C28/I2 treated with miR-130b-5p inhibitor (Figure 4G and H and Figure S4C and D). Taken together, with the overexpression of miR-130b-5p, ECM expression and autophagy flux decreased, whereas the senescence marker increased, conversely, ECM expression and autophagy flux increased, whereas the senescence marker decreased while miR-130b-5p was inhibited.

To further investigate the interaction between circPLXNC1 and miR-130b-5p, we designed circPLXNC1 luciferase plasmid (WT) and a mutant plasmid (Mut). HEK293T cells were separately treated with different circPLXNC1 luciferase plasmid and mimic-miR-130b-5p. The results of luciferase assay showed a markedly stronger luciferase intensity in the circPLXNC1 mutant group compared with the WT group, indicating that miR-130b-5p can directly bind to circPLXNC1 (Figure 4I). qRT-PCR showed that the expression of circPLXNC1 decreased after treated with mimic-miR-130b-5p in C28/I2, while increased after treated with inhibitor-miR-130b-5p (Figure S4G and H). RIP assays were used to detect the function of Ago2 between circPLXNC1 and miR-130b-5p, which revealed that circPLXNC1 could be preferentially pulled down after the overexpression of miR-130b-5p (Figure 4J). These findings revealed the direct interaction between miR-130b-5p and circPLXNC1.

### miR-130b-5p directly targets ATG7

**Figure 5:**
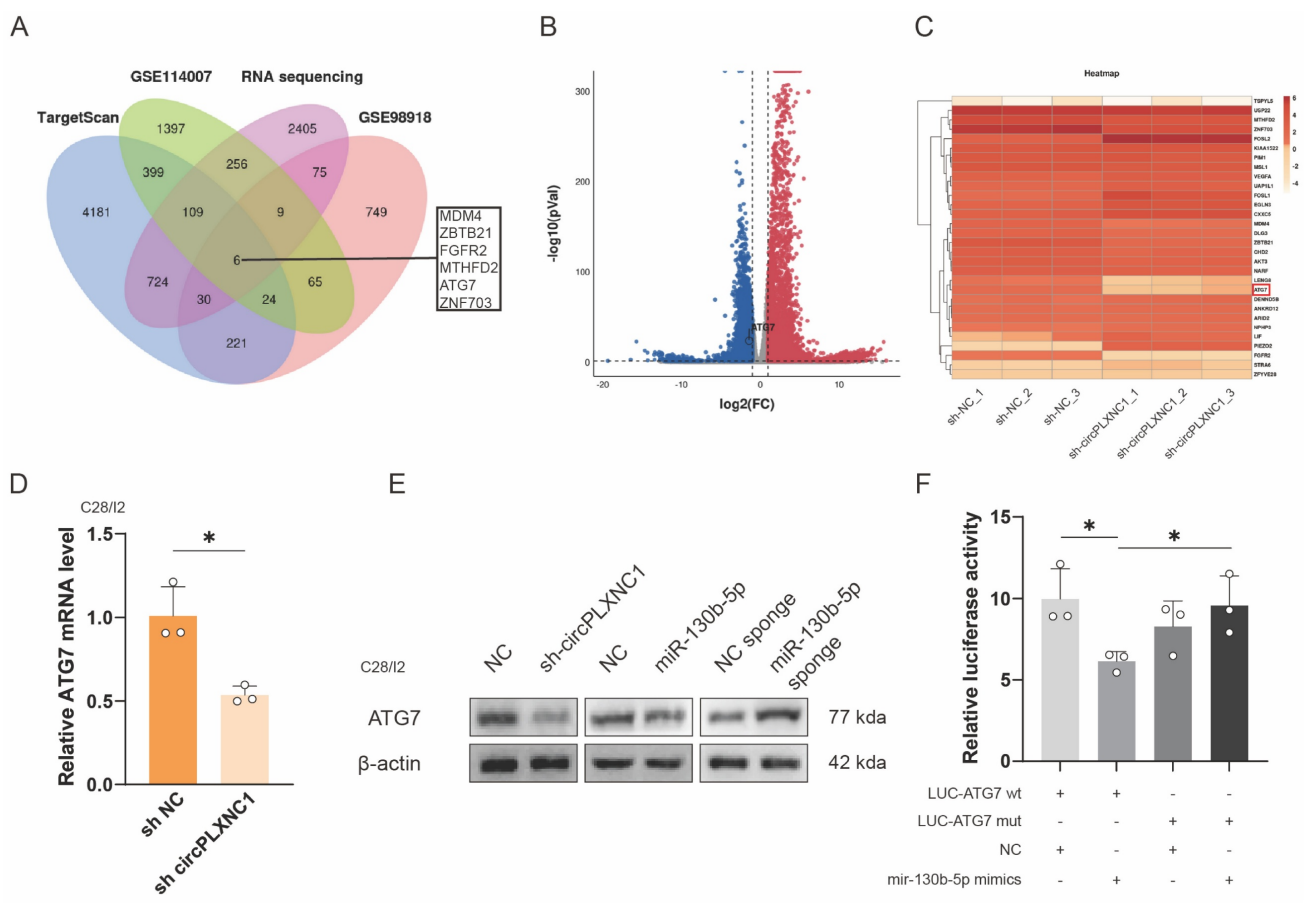
miR-130b-5p directly targets ATG7. (A) Schematic diagram exhibiting the overlap between the downregulated mRNAs identified from the RNA-seq data (Log2(sh-circPLXNC1/NC) < -0.5, p < 0.01) and target gene analyses of miR-130b-5p using the TargetScan database and two OA related database, including GSE114007 and GSE98918. (B) Volcano plot of RNA-seq of C28/I2 treated with sh-circPLXNC1 or sh-NC. (C) Heatmap of circRNA from C28/I2 treated with sh-circPLXNC1 or sh-NC. (D) mRNA expression levels of ATG7 in C28/I2 treated with sh-circPLXNC1 (n=3) or sh-NC (n=3). (E) Western blot detection of ATG7 in C28/I2 treated with sh-circPLXNC1 (n=3), sh-NC (n=3), mimic-miR-130b-5p (n=3), mimic-NC (n=3), miR-130b-5p sponge (n=3) or NC sponge (n=3). (F) HEK293T cells were cotransfected with miR-130b-5p (or NC) and luciferase reporter constructs containing wild-type (WT) or mutated ATG7 3'-UTR. The relative luciferase activity is demonstrated in the histogram. The data are representative of three independent experiments (E). *p<0.05, **p<0.01, ***p<0.001, ****p<0.0001, mean ± SD, two-tailed t test.

To further investigate the function of miR-130b-5p and circPLXNC1, we used the online software TargetScan, two OA-related databases, GSE98918 and GSE114007, and a RNA sequence about C28/I2 treated with sh circPLXNC1 or sh NC to screen potential target genes. 6 genes were obtained from the screen (Figure 5A-C). In addition, qRT-PCR was used to verified the expression change of those 6 circRNAs. Among them, 3 genes changed significantly after treatment with IL-1β, while 2 genes changed significantly after treatment with rapamycin. After the intersection of those two results, the screening result ATG7 was obtained (Figure S5A and B). And the knockdown results of ATG7 in C28/I2 was evaluated by qRT-PCR (Figure S5C). What's more, qRT-PCR was used to detected the expression of ATG7 in C28/I2 treated with sh circPLXNC1 and primary cultured chondrocytes isolated from WT C57BL/6 (B6) mice treated with si mmu-circPLXNC1 (Figure 5D and S5D). Notably, the protein expression of ATG7 decreased in C28/I2 treated with sh circPLXNC1 or mimic-miR-130b-5p, but increased in C28/I2 treated with miR-130b-5p inhibitor (Figure 5E and S5F). These results indicated that the expression of ATG7 increased when circPLXNC1 was upregulated or miR-130b-5p was downregulated, and decreased when circPLXNC1 was downregulated or miR-130b-5p was upregulated.

To further investigate the interaction between ATG7 and miR-130b-5p, we designed ATG7 luciferase plasmid (WT) and a mutant plasmid (Mut). HEK293T cells were separately treated with different ATG7 luciferase plasmid and mimic-miR-130b-5p. The results of luciferase assay showed a markedly stronger luciferase intensity in the ATG7 mutant group compared with the WT group, indicating that miR-130b-5p can directly bind to ATG7 (Figure 5F). In addition, qRT-PCR showed that the expression of miR-130b-5p decreased after overexpression ATG7 in C28/I2, which is not as significant as that of miR-130b-5p on the change in ATG7 expression (Figure S5E).

### Overexpressing circPLXNC1 or ATG7 partially alleviated senescence and OA progression in the aging mouse model and the DMM mouse model

**Figure 6:**
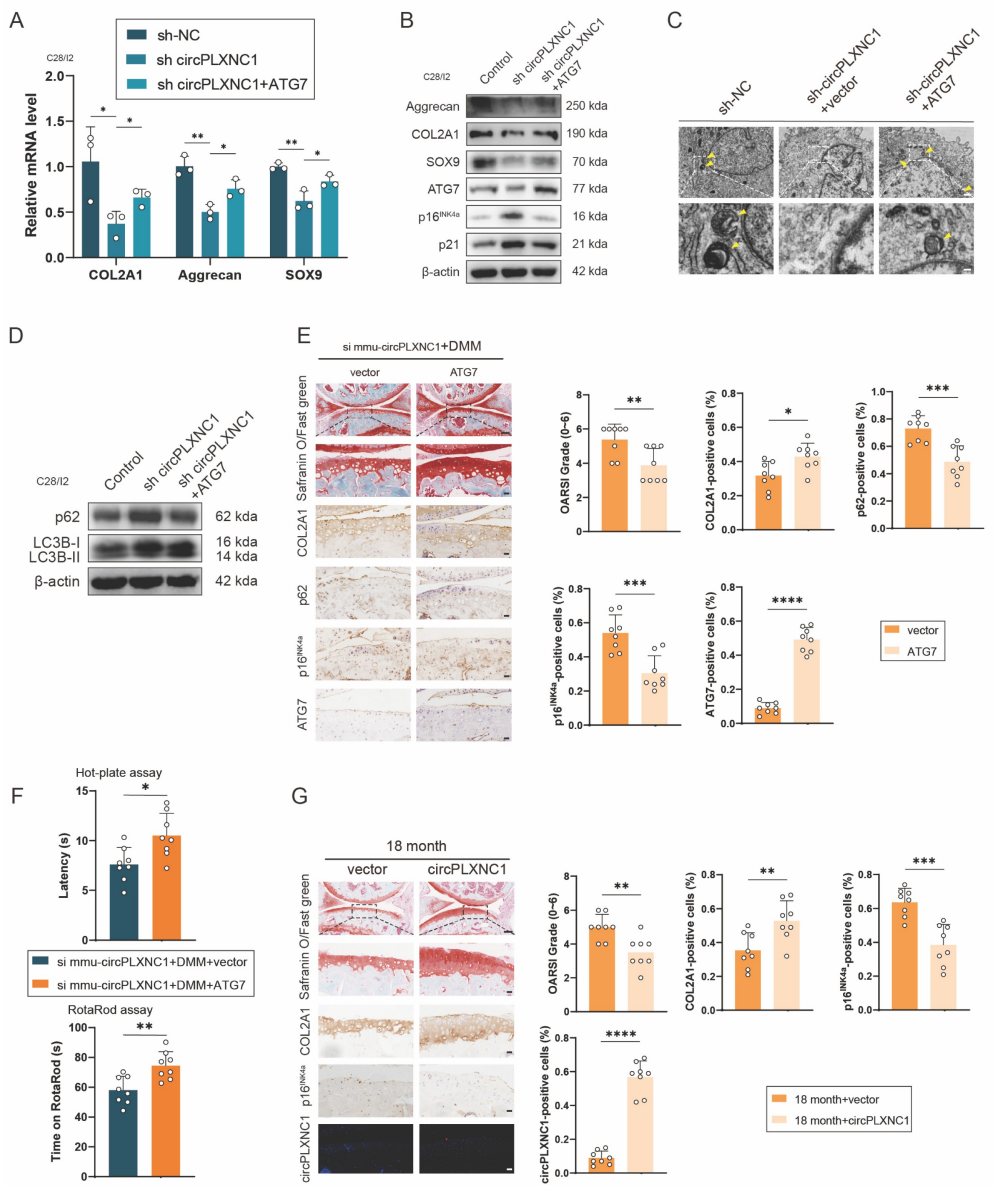
Overexpressing circPLXNC1 or ATG7 partially alleviated senescence and OA progression in the aging mouse model and the DMM mouse model. (A) mRNA expression levels of COL2A1, Aggrecan and SOX9 in C28/I2 cultured and transfected with sh-circPLXNC1 (or sh-NC) and ATG7 (or vector). (B) Western blot detection of COL2A1, Aggrecan, SOX9, p16^INK4a^, p21 and ATG7 in C28/I2 cultured and transfected with sh-circPLXNC1 (or sh-NC) and ATG7 (or vector). (C) The representative electron microscopy images of C28/I2 treated with sh-circPLXNC1 (or sh-NC) and ATG7 (or vector). Yellow arrowheads indicate autophagosomes. Scale bars, 5 μm and 1 μm. (D) Western blot detection of p62 and LC3B in C28/I2 cultured and transfected with sh-circPLXNC1 (or sh-NC) and ATG7 (or vector). (E) Safranin O/Fast Green staining and immunohistochemistry (COL2A1, p62, p16^INK4a^ and ATG7) of the knee joints of mice that underwent DMM and treated with si mmu-circPLXNC1 articular injection. Articular injection of ATG7 adeno-associated virus (AAV) or vector was administered. n=8 per group. Scale bars, 100 μm and 20 μm. (F) The hot plate and rotarod test results of mice that underwent DMM and treated with si mmu-circPLXNC1 articular injection. Articular injection of ATG7 AAV or vector was administered. (G) Safranin O/Fast Green staining, immunohistochemistry (COL2A1 and p16^INK4a^) and RNA fluorescence *in situ* hybridization (FISH) of the knee joints of 18-month old mice. Articular injection of circPLXNC1 AAV or vector was administered. n=8 per group. Scale bars, 100 μm and 20 μm. The data are representative of three independent experiments (B and D). *p<0.05, **p<0.01, ***p<0.001, ****p<0.0001, mean ± SD, two-tailed t test.

We designed an ATG7-overexpressing lentiviral plasmid and transfected it into the C28/I2 cells, which were transfected with sh circPLXNC1 or sh NC, to confirm whether it could suppress OA and senescence progression. Western blotting and qRT‒PCR analysis of C28/I2 (transfected with sh circPLXNC1) transfected with the ATG7 lentivirus or vector revealed that the transcript and protein expression of anabolic enzymes, including Aggrecan, COL2A1, and SOX9, increased in response to the overexpression of ATG7 (Figure 6A and B and S6A). In contrast, the levels of senescence marker, p16^INK4a^ and p21, decreased significantly after treatment with the ATG7 lentivirus (Figure 6B and S6A). In the results of TEM, the number of autophagosomes increased in C28/I2 treated with ATG7 lentivirus (Figure 6C). Similarly, after treated with ATG7 lentivirus, protein expression of autophagy markers, p62, decreased and the ratio of LC3B-II to LC3B-I increased in C28/I2 (Figure 6D and S6B). These findings suggested that with the knockdown of circPLXNC1, anabolic enzyme expression and autophagy flux decreased, whereas the senescence marker increased. In addition, after DMM surgery, ATG7 adeno-associated virus (AAV) was injected into the articular cavity of wild-type mice, which were also injected with si mmu-circPLXNC1. OARSI scores were lower in the group treated with ATG7 AAV than in the group treated with vector, as determined by Safranin O staining (Figure 6E). What's more, IHC revealed that the expression of COL2A1 and ATG7 was greater in the group treated with ATG7 AAV, whereas the expression of p16^INK4a^ and p62 was lower, indicating that the increase in the expression of anabolic enzymes and autophagy flux, and the decrease in the expression of senescence markers were related to the increase in ATG7 in DMM-induced OA model mice treated with si mmu-circPLXNC1 (Figure 6E). Furthermore, mice treated with ATG7 AAV were less sensitive to pain and had a lower degree of mobility impairment in the hot plate test and rotarod test (Figure 6F).

To detect the function of circPLXNC1 overexpression in aging, we injected circPLXNC1 AAV or vector into the articular cavity of 18-month-old mice. Safranin O staining showed that OARSI scores were lower in the group treated with circPLXNC1 AAV than in the group treated with vector (Figure 6G). In addition, IHC revealed that the expression of COL2A1 was greater in the group treated with circPLXNC1 AAV, whereas the expression of p16^INK4a^ was lower, indicating that the increase in the expression of anabolic enzymes and the decrease in the expression of senescence markers were related to the increase in circPLXNC1 in aging mice treated with circPLXNC1 AAV (Figure 6G).

**Figure 7:**
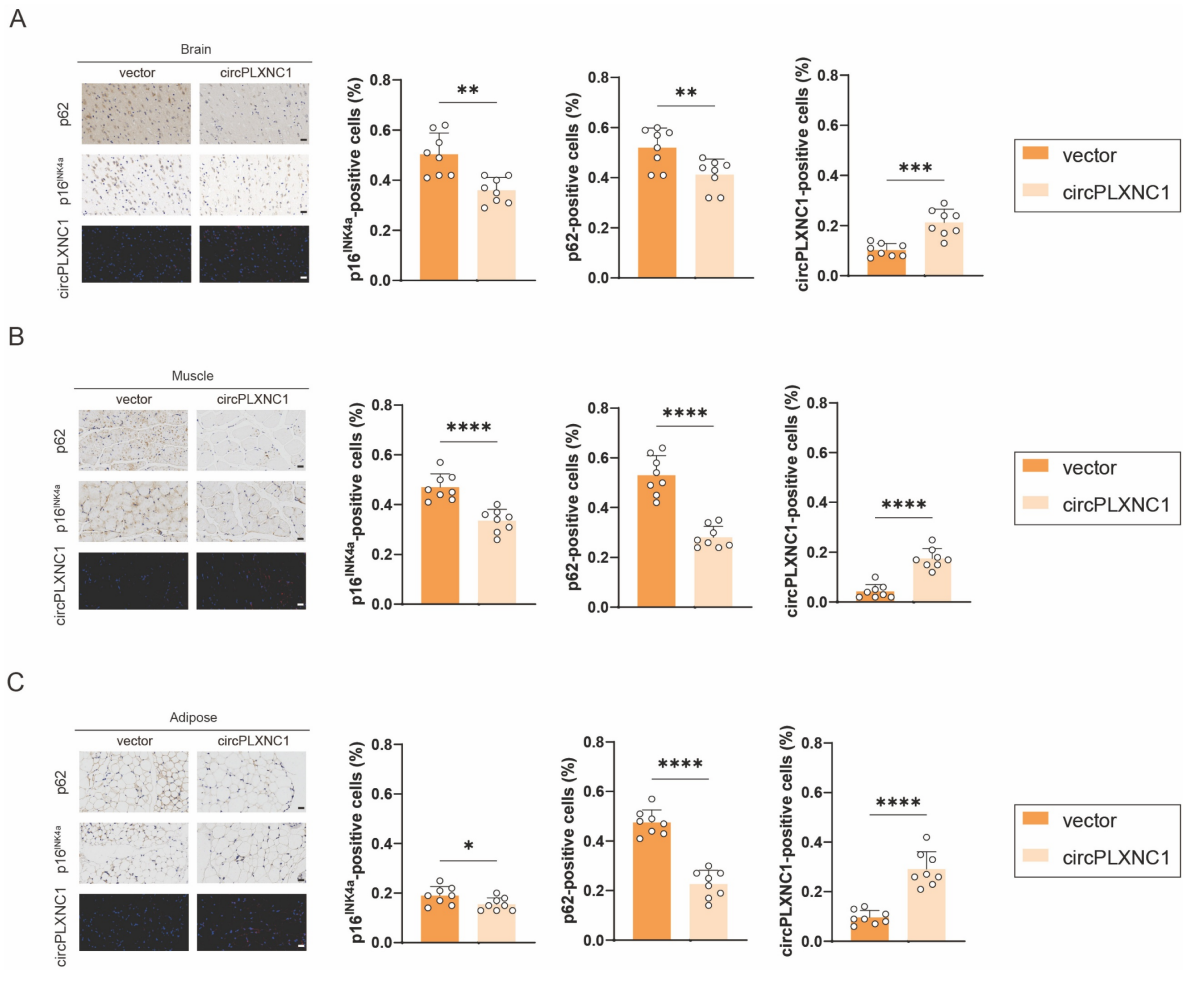
Overexpressing circPLXNC1 partially alleviated senescence in the aging mouse model. (A) Immunohistochemistry (p62 and p16^INK4a^) and RNA fluorescence *in situ* hybridization (FISH) of the brain of 18-month-old mice. Tail vein injection of circPLXNC1 AAV or vector was administered. n=8 per group. Scale bars, 20 μm. (B) Immunohistochemistry (p62 and p16^INK4a^) and RNA fluorescence *in situ* hybridization (FISH) of the muscle tissue of 18-month-old mice. Tail vein injection of circPLXNC1 AAV or vector was administered. n=8 per group. Scale bars, 20 μm. (C) Immunohistochemistry (p62 and p16^INK4a^) and RNA fluorescence *in situ* hybridization (FISH) of the adipose tissue of 18-month-old mice. Tail vein injection of circPLXNC1 AAV or vector was administered. n=8 per group. Scale bars, 20 μm. *p<0.05, **p<0.01, ***p<0.001, ****p<0.0001, mean ± SD, two-tailed t test.

To further detect the function of circPLXNC1 in aging, tail vein injection was used to deliver circPLXNC1 AAV into 18-month-old mice. FISH results verified that circPLXNC1 expression increased with the use of AAV in brain, muscle and adipose (Figure 7A-C). In addition, IHC revealed that the expression of p16^INK4a^ and p62 was lower after treating with circPLXNC1 AAV in those organ and tissue, which indicated that circPLXNC1 could alleviate the aging of multiple organs and tissues and increase the autophagy flux (Figure 7A-C). These findings suggested that circPLXNC1 and ATG7 are potential therapeutic targets for senescence and OA.

## Discussion

Aging, as a complex programmed process, affects different organs and tissues, while involving changes of characteristic phenotypes.^52^ In this study, we demonstrated the key role of circular RNA circPLXNC1 in aging process of multiple organs and tissues, including brain, muscle, adipose, and cartilage, by regulating ATG7 to affect autophagy. Previous studies have detailed the effect of senescence in several tissues, like increased SASP, disordered metabolism, altered autophagy and etc.^8^ For instance, increased activity of SA-β-gal and expression of cycling-dependent kinase inhibitors, such as p16^INK4A^ and p21, was found in Alzheimer's patients' senescent neurons and OA patients' senescent chondrocytes.^2^, ^25^

Similarly, autophagy flux decreased in aging associated neurodegenerative diseases, and was modulated by METTL3-mediated m6A modification of ATG7.^16^, ^53^ Moreover, senescent chondrocytes of OA patients are accompanied with disordered lipid metabolism, regulated by circRREB1 through FASN post-translational modifications, as well as disordered amino acid metabolism, affected by 10-hydroxy-2-decenoic acid targeting aspartyl β hydroxylase.^21^, ^54^ Although with such mechanisms have been uncovered, senescence, as the most familiar yet least well-understood aspect of biology, still requires deeper exploration of therapeutic targets and strategies.

Our study further explores mechanisms of senescence in OA. OA, the most common age-related chronic joint disease affecting more than 240 million people worldwide, is characterized by degenerative joint damage.^17^ In senescent chondrocytes, Aggrecan and COL2A1 expression and autophagy flux decreased, on the other hand, SASP expression and organelle destruction increased.^55^ Previous work from others has demonstrated that signaling pathways, such as TGF-β/SMAD2/3 signaling, Notch signaling and JAK3/STAT5 signaling, play key role in regulating senescence in OA.^24^, ^26^, ^28^ For instance, the regulation of Notch signaling to OA is affected by the enhancement of clathrin-mediated endocytosis leading by the deficiency of MYL3, while chondrocyte senescence leading by inhibiting JAK3/STAT5 signaling could be inhibited by Sirt6 24, 26. What's more, Semane 6D (SEMA6D), as a cardiovascular neuroeffector that helps maintain chondrocyte homeostasis, its overexpression can regulate ECM metabolism and chondrocyte hypertrophy by inhibiting the AGT/AGTR1a/IL-1β axis.^56^ Similarly, Gossypol acetic acid (GAA), a medicinal form of Gossypol, a natural phenolic compound isolated from cotton seeds, regulates ECM-related factors in chondrocytes and alleviates the ferroptosis in chondrocytes by inhibiting GPX4 methylation in OA.^57^ Meanwhile, the transcriptome-wide association study (TWAS) was used to identified high-confidence candidate genes of OA, and both *in vitro* and *in vivo* experiments verified that the genes had high activity and enriched expression in OA.^58^

Autophagy flux decreasing is a one of the signs of chondrocytes senescence.^55^ Rapamycin serves as a potent activator of autophagy to induce autophagic processes.^59^ In mice, Rapamycin injection has been proved to alleviate the OA progression.^60^ However, due to the low solubility of Rapamycin in the joint cavity and the need for high frequency administration due to lymphatic clearance, biomaterial technology must be used, which requires high cost and further exploration.^61^, ^62^ For instance, poly (lactic-co-glycolic acid) nanoparticles loaded with Rapamycin was used in the treatment of OA to address the shortcomings of Rapamycin.^63^ Compared with the direct use of Rapamycin, the structure of circRNA was stable, and the frequency of administration using AAV was significantly reduced. In addition, our RNA sequencing results revealed that the use of Rapamycin can affect multiple targets, compared with circRNA, which can more accurately regulate downstream target genes with fewer possible side effects. In fact, circRNA is already regarded as an essential factor in OA regulation.^31^ Previous studies reported that circPDE4D regulated FGF18 by binding to miR-103a-3p to protect against OA.^35^ Similarly, circPDE4B acted as a scaffold for RIC8A and MID1 to prevent articular cartilage degeneration.^45^ Conversely, circRSU1 promoted the progression of OA by adjusting oxidative stress.^64^ Those studies mainly address on cartilage degeneration, while we focus on aging of cartilage and other organs and tissues besides cartilage degeneration. Here, we validated that circPLXNC1, which was significantly downregulated in OA cartilage, promoted chondrocytes anabolism, including Aggrecan and COL2A1, and inhibited chondrocyte senescence, including SASP expression and decrease of autophagy, by acting as a sponge for miR-130b-5p. Senescence and autophagy in organs and tissues other than cartilage such as brain, muscle, and adipose can also be reversed by circPLXNC1. Besides, other types of RNA also play important roles in OA. For instance, mRNA UTR optimization and chemical modified FGF18 mRNA can infiltrate and deliver deeper than proteins in the cartilage through lipid nanoparticle (LNP), and significantly alleviate the symptoms of OA.^65^ Similarly, long non-coding RNA LncZFHX2 forms a transcription complex with KLF4 and promotes DNA repair in chondrocytes.^66^ In addition, many emerging drugs have been used to treat OA, such as BMP-7, HSA, β-NGF, TGF-β inhibitors, etc., with good effect and few side effects.^67^ Among them, TGF-β inhibitors may be effective in treating arthrofibrosis and can interrupt the driving effect of TGF-β on arthrofibrosis.^68^ More effective treatment strategies for OA requires deeper exploration in the future.

Autophagy regulation has become an important means to regulate chondrocyte senescence.^55^ The ablation of autophagy-indispensable ATG5 gene was associated with an increased cell death and facilitated age-related OA.^39^ Another autophagy-indispensable gene, ATG7, whose m6A modification was regulated by METTL3, affected cellular senescence and osteoarthritis progression.^16^ What's more, ATG7 was used for secretion and exocytosis in autophagy, including targeting intracellular cargo to lysosomes, plasma membrane or extracellular environment.^43^ We determined that circPLXNC1 expression decreased in senescent chondrocytes, and could be reversed by increasing the level of chondrocyte autophagy flux, which was promoted by Rapamycin. Our experiments further showed that circPLXNC1 affected autophagy flux through regulating ATG7 by binding to miR-130b-5p.

**Figure 8:**
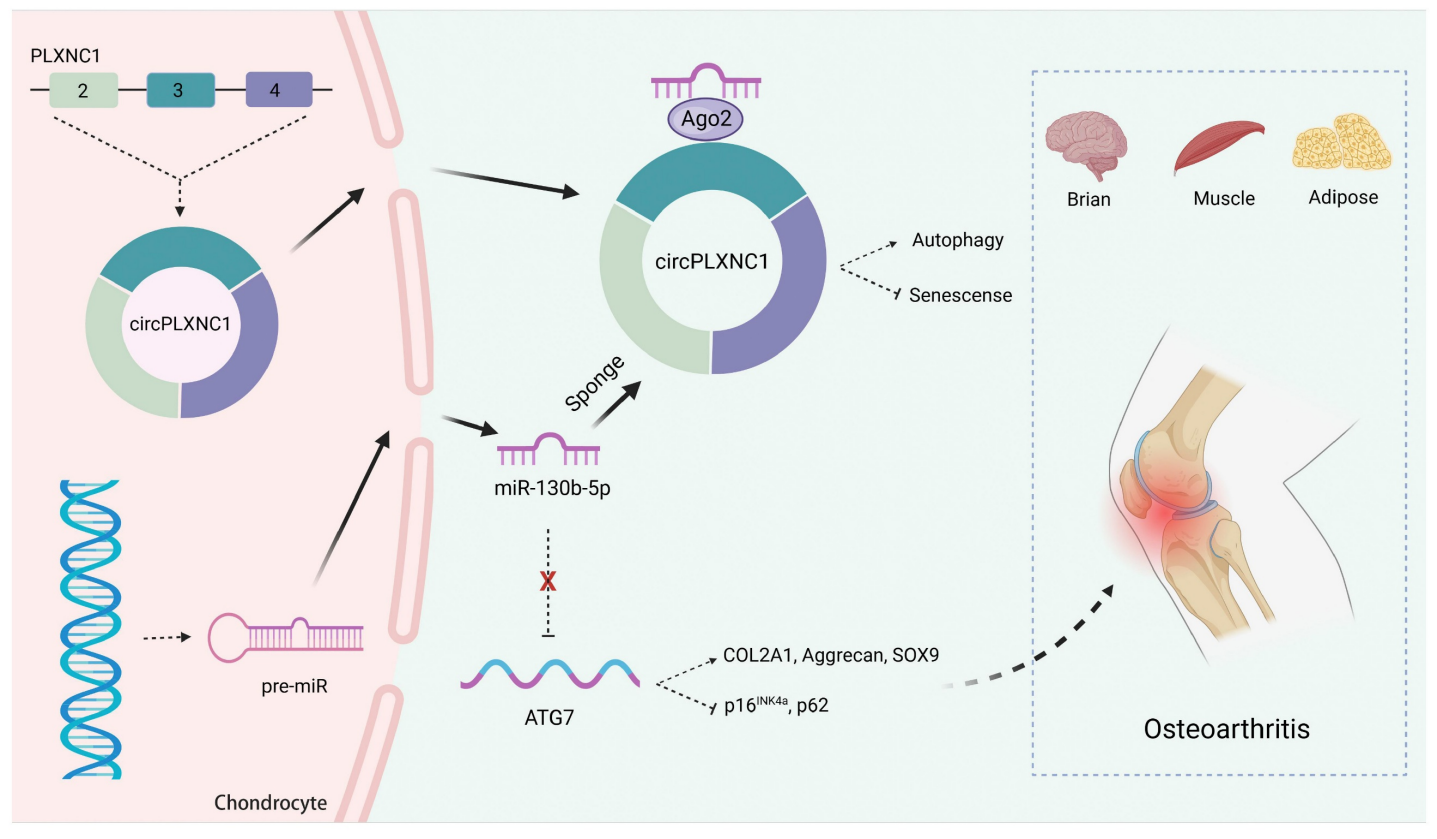
Schematic of the working hypothesis for circPLXNC1. CircPLXNC1 acts as a sponge for miR-130b-5p to directly regulate the expression of autophagy associated enzyme ATG7. In agreement with the function of ATG7 AAV that alleviates OA, the administration of circPLXNC1 AAV exerts similar function to alleviate OA and acts as a inhibitor of senescence to attenuate the senescence of multiple organs and tissues, including cartilage, brain, muscle and adipose.

In conclusion, we described a new circular RNA mechanism in senescence and autophagy. We demonstrated that circPLXNC1 could regulate ATG7, which is essential for autophagy and cytoplasmic to vacuole transport, to affect autophagy in senescent chondrocytes by binding to miR-130b-p. In OA therapy, we found overexpression of circPLXNC1 and ATG7 could protect cartilage from senescence and OA, which is better than the way of using Rapamycin directly. Furthermore, circPLXNC1 overexpression alleviated aging of other organs and tissues of mice, including brain, muscle and adipose, by injecting into the tail vein (Figure 8). However, the mechanisms of circPLXNC1 in regulating autophagy and senescence in those organs and tissues need further exploration. Cumulatively, circPLXNC1 and ATG7 are potential targets for alleviating aging, especially for age-associated OA therapy.

## Supplementary Material

Supplementary figures.

## Abbreviations

OA: Osteoarthritis
AAV: adeno-associated virus
ECM: extracellular matrix
IL-1β: Interleukin-1β
TNF-α: tumor necrosis factor α
IHC: Immunohistochemistry
OARSI: Osteoarthritis Research Society International
DMM: Destabilization of the medial meniscus
RIP: RNA binding protein immunoprecipitation assay
SASP: senescence-associated secretory phenotype
shRNA: small hairpin RNA
H&E: Hematoxylin-Eosin staining
CCK-8: Cell Counting Kit-8
