# Supplementary material for: Circular RNA circPLXNC1 functions as an inhibitor of senescence by regulating autophagy in osteoarthritis
Source: Int J Biol Sci. 2026 Jan 1;22(1):346–64. doi: 10.7150/ijbs.114757 (PMC12681868; doi:10.7150/ijbs.114757)
Supplement: Supplementary file 1 — Supplementary figures. [file ijbsv22p0346s1.pdf]

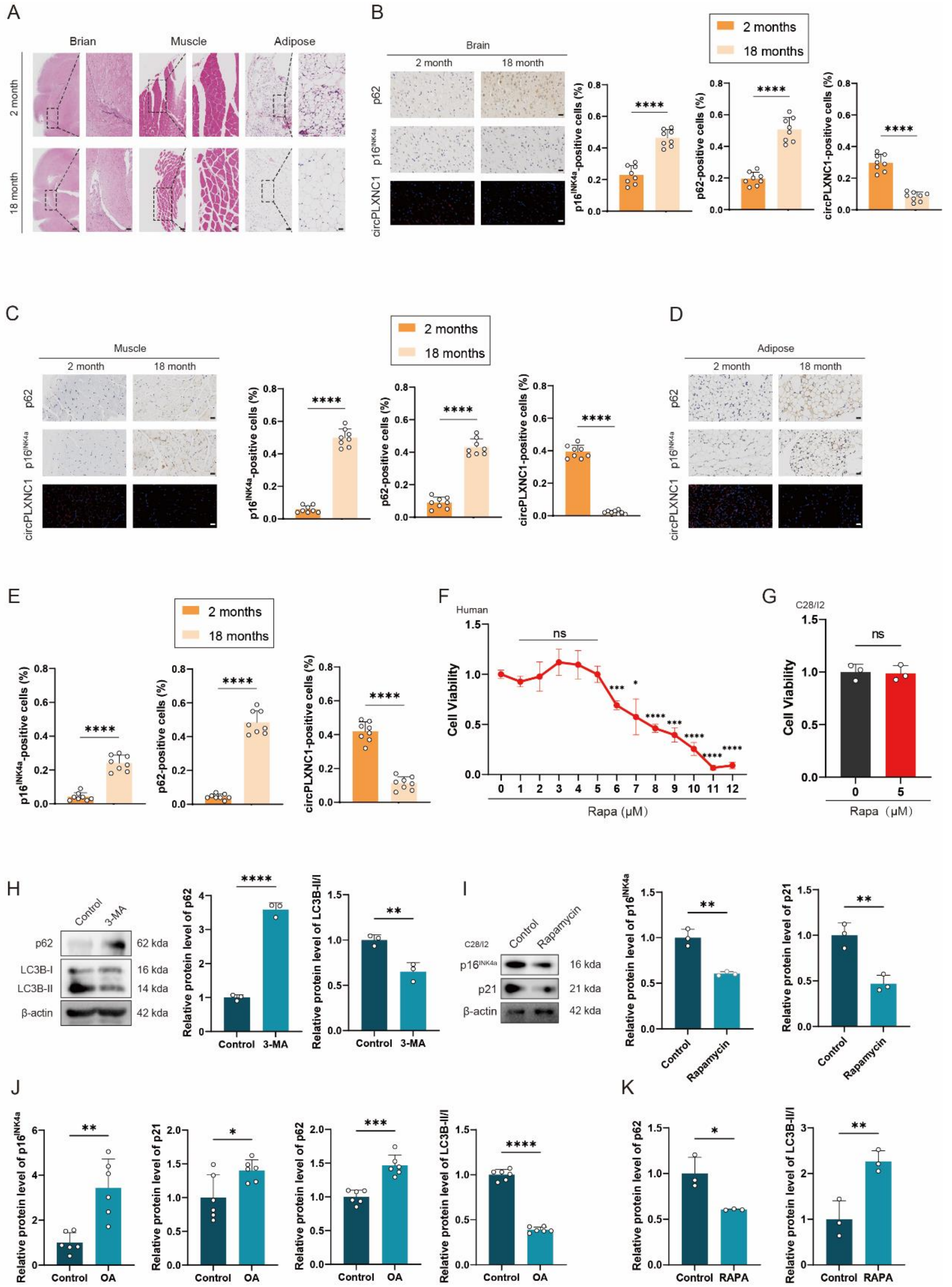

Figure S1 SASP increases in multiple organs and tissues of aging mouse model with impaired autophagy. (A) H&E staining of 2-month old and 18-month old mice. Scale bars of brain and adipose, 100 μm and 20 μm. Scale bars of muscle, 500 μm and 100 μm. (B) Immunohistochemistry (p62 and $p16^{INK4a}$) and RNA fluorescence in situ hybridization (FISH) of the brain of 2-month old and 18-

month old mice. n=8 per group. Scale bars, 20 μm. (C) Immunohistochemistry (p62 and $p16^{INK4a}$) and RNA fluorescence in situ hybridization (FISH) of the muscle tissue of 2-month old and 18-month old mice. n=8 per group. Scale bars, 20 μm. (D) and (E) Immunohistochemistry (p62 and $p16^{INK4a}$) and RNA fluorescence in situ hybridization (FISH) of the adipose tissue of 2-month old and 18-month old mice. n=8 per group. Scale bars, 20 μm. (F) CCK8 detection of cell viability of human chondrocytes treated with Rapamycin in different concentration. (G) CCK8 detection of cell viability of C28/I2 treated with Rapamycin or not. (H) Western blot detection of p62 and LC3B in C28/I2 treated with 3-MA or not. (I) Western blot detection of $p16^{INK4a}$ and p21 in C28/I2 treated with Rapamycin or not. (J) Quantification of western blot detection of $p16^{INK4a}$, p21, p62 and LC3B expression in human primary chondrocytes from the damaged cartilage group or the undamaged cartilage group. (K) Quantification of western blot detection of p62 and LC3B in C28/I2 treated with Rapamycin or not. The data are representative of three independent experiments (H and I). *$p<0.05$, **$p<0.01$, ***$p<0.001$, ****$p<0.0001$, mean ± SD, two-tailed t test.

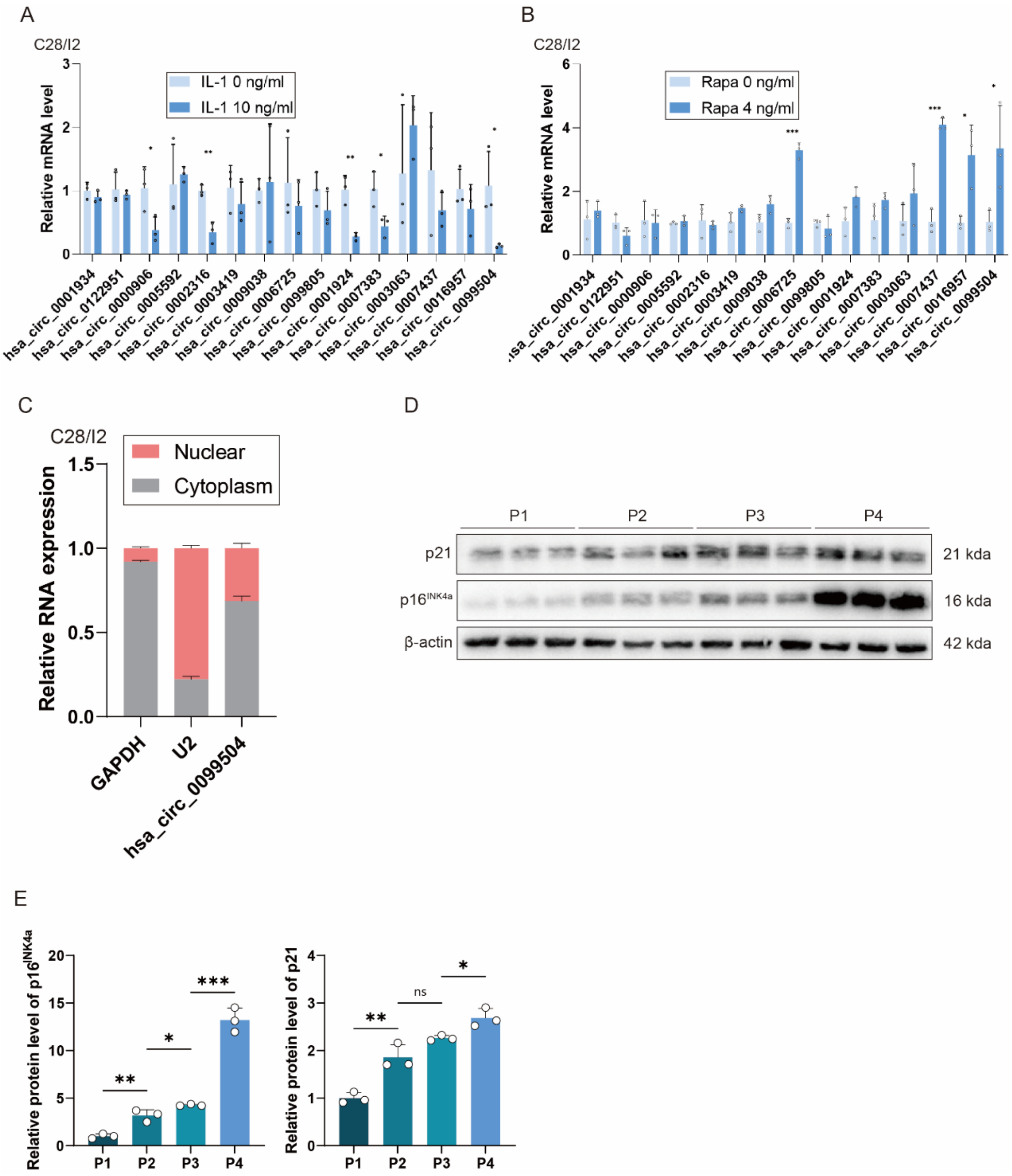

Figure S2 Screening circRNAs associated with OA and autophagy. (A) The mRNA expression level of 15 circRNAs treated with IL-1β or not in C28/I2. (B) The mRNA expression level of 15 circRNAs treated with Rapamycin or not in C28/I2. (C) Expression of circPLXNC1 assessed by RT-qPCR in the nuclear and cytoplasmic fractions of C28/I2. (D) Western blot detection of $p16^{INK4a}$ and p21 in human primary chondrocytes cultured to P1-4 generation. (E) Quantification of western blot detection of $p16^{INK4a}$ and p21 in human primary chondrocytes cultured to P1-4 generation. The data are representative of three independent experiments (D). $*p<0.05$, $**p<0.01$, $***p<0.001$,

****p<0.0001, mean ± SD, two-tailed t test.

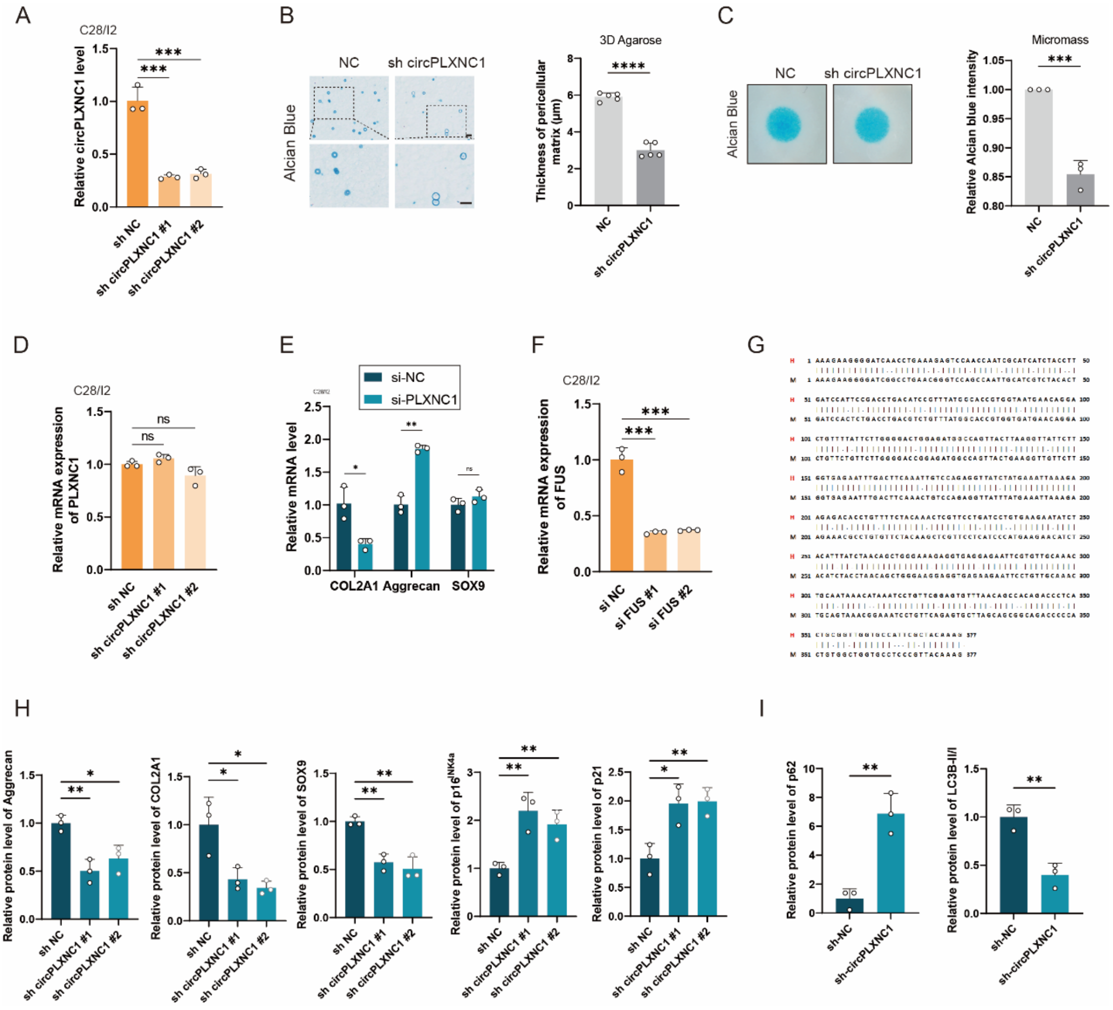

Figure S3 Effect of sh-circPLXNC1 on ECM. (A) The mRNA expression level of circPLXNC1 in C28/I2 treated with two types of sh-circPLXNC1 or sh-NC. (B) Chondrogenic matrix deposition (Alcian Blue staining) of C28/I2, treated with sh-circPLXNC1 or sh-NC, determined by micromass culture and quantified by ImageJ software. (C) 3D agarose culture of C28/I2 (Alcian blue staining) showing the thickness of pericellular matrix. Scale bar, 20 μm. (D) The mRNA expression level of PLXNC1 in C28/I2 treated with two types of sh-circPLXNC1 or sh-NC. (E) The mRNA expression level of COL2A1, Aggrecan and SOX9 in C28/I2 treated with si-PLXNC1 or si-NC. (F) The mRNA

expression level of FUS in C28/I2 treated with two types of si-FUS or si-NC. (G) Sequence alignment of human circPLXNC1 and the mouse circPLXNC1 homolog. (H) Quantification of western blot detection of COL2A1, Aggrecan, SOX9, p16^{INK4a} and p21 in C28/I2 treated with sh-circPLXNC1 or sh-NC. (I) Quantification of western blot detection of p62 and LC3B in C28/I2 treated with sh-circPLXNC1 or sh-NC. *p<0.05, **p<0.01, ***p<0.001, ****p<0.0001, mean ± SD, two-tailed t test.

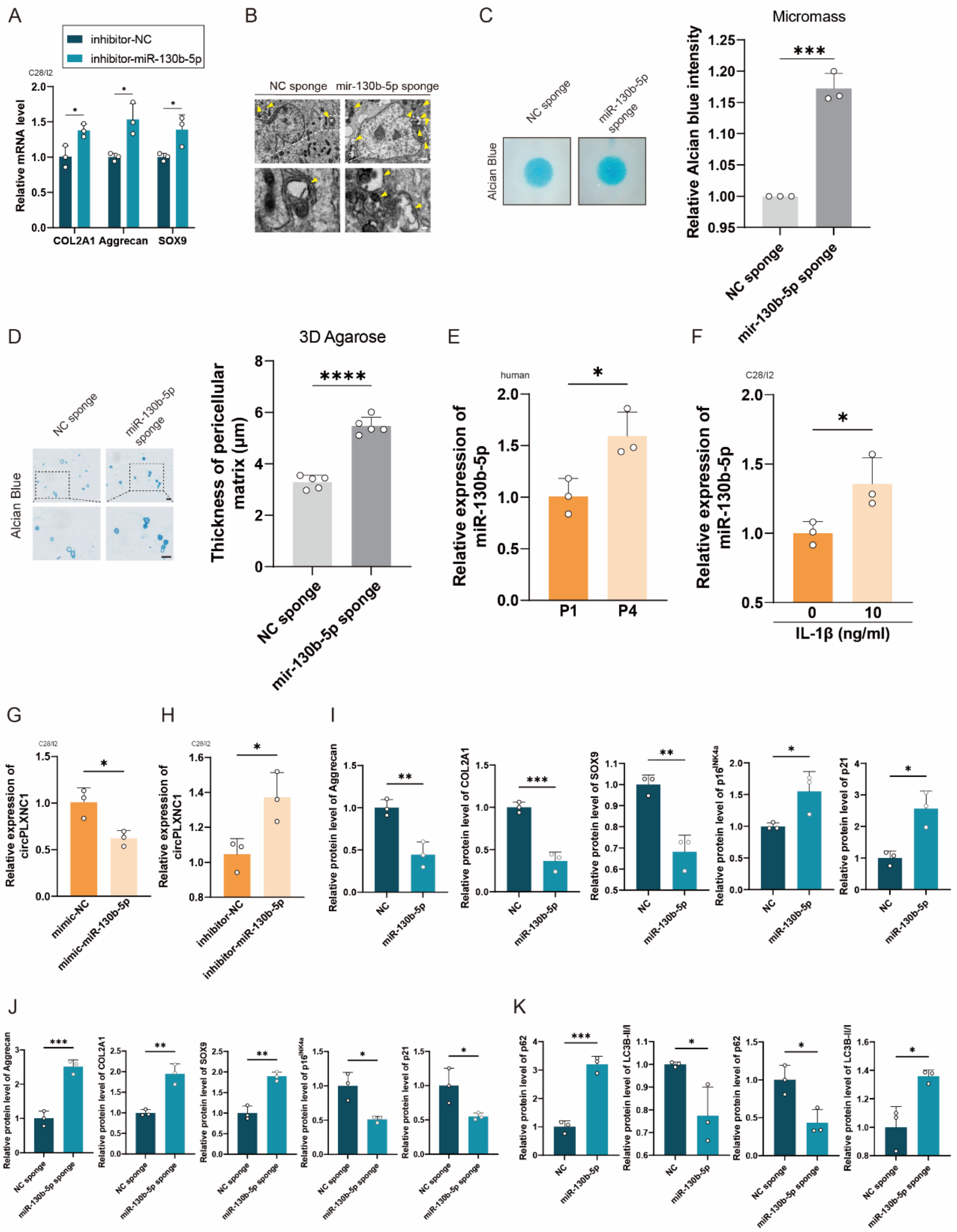

Figure S4 Effect of miR-130b-5p on ECM and autophagy. (A) The mRNA expression level of COL2A1, Aggrecan and SOX9 in C28/I2 treated with inhibitor-miR-130b-5p or inhibitor-NC. (B) The representative electron microscopy images of C28/I2 treated with inhibitor-miR-130b-5p or inhibitor-NC. Yellow arrowheads indicate autophagosomes. Scale bars, 5 μm and 1 μm. (C) Chondrogenic matrix deposition (Alcian Blue staining) of C28/I2, treated with inhibitor-miR-130b-

5p or inhibitor-NC, determined by micromass culture and quantified by ImageJ software. (D) 3D agarose culture of C28/I2 (Alcian blue staining) showing the thickness of pericellular matrix. (E) The relative level of miR-130b-5p in human primary chondrocytes cultured to P1 or P4 generation. (F) The relative level of miR-130b-5p in C28/I2 treated with IL-1β or not. (G) The mRNA expression level of circPLXNC1 in C28/I2 treated with mimic-miR-130b-5p or mimic-NC. (H) The mRNA expression level of circPLXNC1 in C28/I2 treated with inhibitor-miR-130b-5p or inhibitor-NC. (I) Quantification of western blot detection of COL2A1, Aggrecan, SOX9, p16^{INK4a} and p21 in C28/I2 treated with mimic-miR-130b-5p and mimic-NC. (J) Quantification of western blot detection of COL2A1, Aggrecan, SOX9, p16^{INK4a} and p21 in C28/I2 treated with miR-130b-5p sponge or NC sponge. (K) Quantification of western blot detection of p62 and LC3B in C28/I2 treated with mimic-miR-130b-5p, mimic-NC, miR-130b-5p sponge or NC sponge. Scale bar, 20 μm. *p<0.05, **p<0.01, ***p<0.001, ****p<0.0001, mean ± SD, two-tailed t test.

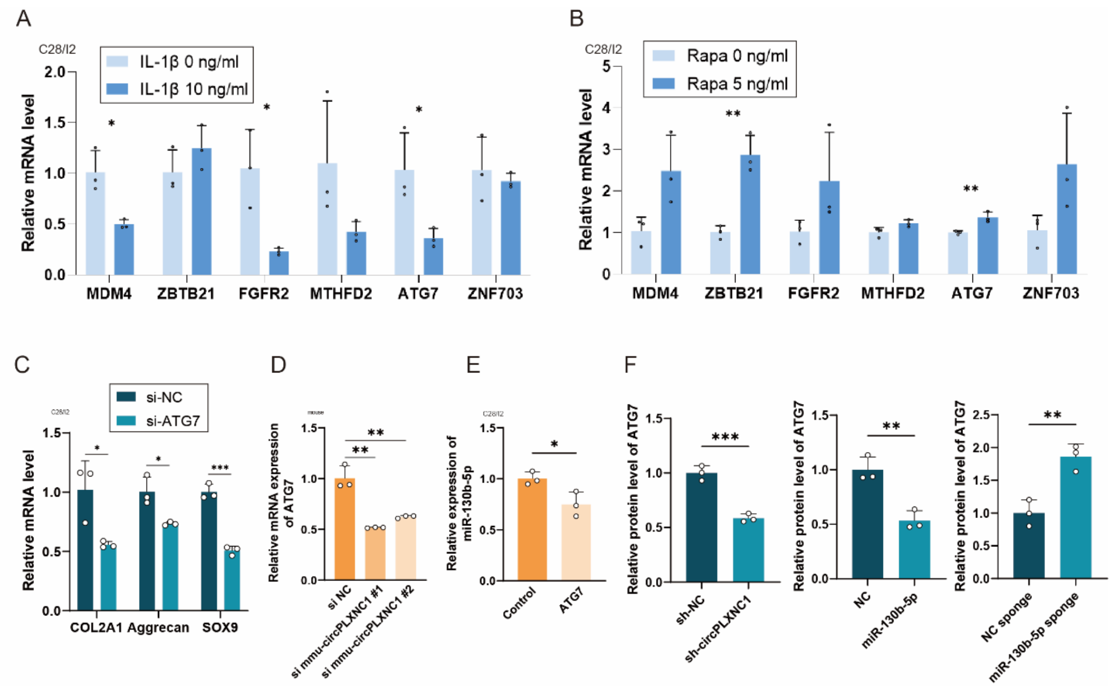

Figure S5 Screen target gene of miR-130b-5p. (A) The mRNA expression level of 6 target genes treated with IL-1β or not in C28/I2. (B) The mRNA expression level of 6 target genes treated with Rapamycin or not in C28/I2. (C) The mRNA expression level of COL2A1, Aggrecan and SOX9 in C28/I2 treated with si-ATG7 or si-NC. (D) The mRNA expression level of ATG7 in primary cultured chondrocytes isolated from WT C57BL/6 (B6) mice treated with si mmu-circPLXNC1 or si NC. (E) The relative level of miR-130b-5p in C28/I2 cultured and transfected with ATG7 or vector. (F) Quantification of western blot detection of ATG7 in C28/I2 treated with sh-circPLXNC1, sh-NC, mimic-miR-130b-5p, mimic-NC, miR-130b-5p sponge or NC sponge. *$p<0.05$, **$p<0.01$, ***$p<0.001$, ****$p<0.0001$, mean ± SD, two-tailed t test.

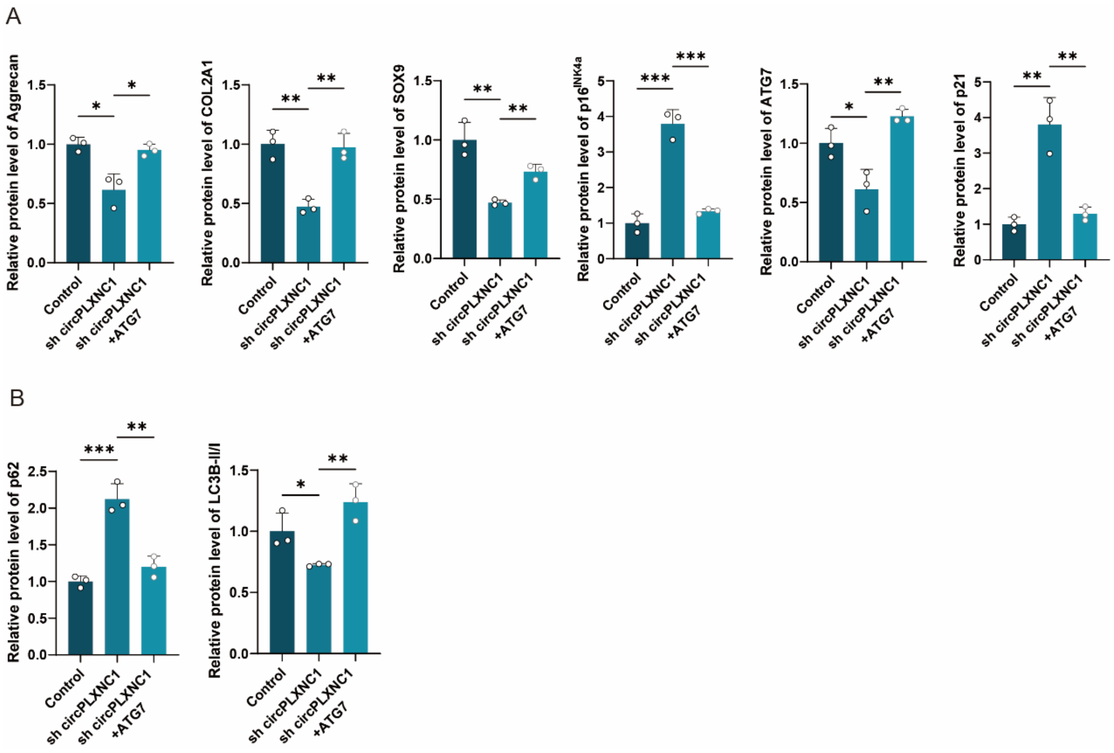

Figure S6 Overexpressing ATG7 partially alleviated senescence and OA progression. (A) Quantification of western blot detection of COL2A1, Aggrecan, SOX9, $p16^{INK4a}$, p21 and ATG7 in

C28/I2 cultured and transfected with sh-circPLXNC1 (or sh-NC) and ATG7 (or vector). (A) Quantification of western blot detection of p62 and LC3B in C28/I2 cultured and transfected with sh-circPLXNC1 (or sh-NC) and ATG7 (or vector). *$p<0.05$, **$p<0.01$, ***$p<0.001$, ****$p<0.0001$, mean ± SD, two-tailed t test.
